# Accuracy of Kinovea software in estimating body segment movements during falls captured on standard video: Effects of fall direction, camera perspective and video calibration technique

**DOI:** 10.1371/journal.pone.0258923

**Published:** 2021-10-25

**Authors:** Nataliya Shishov, Karam Elabd, Vicki Komisar, Helen Chong, Stephen N. Robinovitch

**Affiliations:** 1 Department of Biomedical Physiology and Kinesiology, Simon Fraser University, Burnaby, British Columbia, Canada; 2 School of Engineering Science, Simon Fraser University, Burnaby, British Columbia, Canada; 3 School of Engineering, The University of British Columbia, Kelowna, British Columbia, Canada; Tokai University, JAPAN

## Abstract

Falls are a major cause of unintentional injuries. Understanding the movements of the body during falls is important to the design of fall prevention and management strategies, including exercise programs, mobility aids, fall detectors, protective gear, and safer environments. Video footage of real-life falls is increasingly available, and may be used with digitization software to extract kinematic features of falls. We examined the validity of this approach by conducting laboratory falling experiments, and comparing linear and angular positions and velocities measured from 3D motion capture to estimates from Kinovea 2D digitization software based on standard surveillance video (30 Hz, 640x480 pixels). We also examined how Kinovea accuracy depended on fall direction, camera angle, filtering cut-off frequency, and calibration technique. For a camera oriented perpendicular to the plane of the fall (90 degrees), Kinovea position data filtered at 10 Hz, and video calibration using a 2D grid, mean root mean square errors were 0.050 m or 9% of the signal amplitude and 0.22 m/s (7%) for vertical position and velocity, and 0.035 m (6%) and 0.16 m/s (7%) for horizontal position and velocity. Errors in angular measures averaged over 2-fold higher in sideways than forward or backward falls, due to out-of-plane movement of the knees and elbows. Errors in horizontal velocity were 2.5-fold higher for a 30 than 90 degree camera angle, and 1.6-fold higher for calibration using participants’ height (1D) instead of a 2D grid. When compared to 10 Hz, filtering at 3 Hz caused velocity errors to increase 1.4-fold. Our results demonstrate that Kinovea can be applied to 30 Hz video to measure linear positions and velocities to within 9% accuracy. Lower accuracy was observed for angular kinematics of the upper and lower limb in sideways falls, and for horizontal measures from 30 degree cameras or 1D height-based calibration.

## 1. Introduction

Falls are the number one cause of unintentional injury and the number two cause of injury-related deaths world-wide [1,2]. Each year, falls cause 37 million hospital visits, 646,000 fatalities [2], and treatment costs over USD 50 billion globally [3]. Falls cause up to 80% of traumatic brain injuries [4] and 95% of hip fractures [5,6] in older adults. The high prevalence and clinical consequences of falls stress the need for improvements in the design and implementation of strategies for predicting, detecting, and preventing falls and injuries.

Improved understanding of the kinematics of real-life falls should help to inform efforts for injury prevention. For example, information on the movements of the body during falls can help exercise therapists in designing exercise programs for fall prevention [7–10], engineers in creating mobility aids [11–13] and architects and designers in creating safer environments [14–17]. Data on the kinematics of falls can also guide the design of improved sensor-based automatic fall detection systems [18–23]. Furthermore, data on the impact velocities of the body during falls is essential for the design and evaluation of fall protective gear [24–28] and compliant flooring.

Laboratory studies have used 3D motion capture to measure the kinematics of falls in young adults [29–32]. Studies have also examined the accuracy of wearable sensor systems and video-based approaches for detecting falls in the lab environment [21,33,34]. However, the kinematics of real-life falls may be considerably different from those observed in laboratory studies due to differences in environmental and situational factors (including the cause of the fall), and the population of interest.

2D planar video footage of real-life falls is increasingly available [35–38]. Previous studies have found that the frequency content of body movements during falls ranges from 1–10 Hz [32,39,40], which is below the 30 Hz (or frames per second) capture rate common to video surveillance cameras. However, falls often involve complex 3D movements, and validated approaches are required for extracting body segment kinematics from 2D video images. Furthermore, we need to understand how measurement accuracy depends on frame rate, camera orientation, and calibration technique [41]. Among the software packages available for estimating kinematics from planar video, there is growing use of the open-source software package Kinovea (http://www.kinovea.org). When compared to 3D motion capture, Kinovea estimated hip, knee, and ankle angles in the sagittal plane during walking with errors less than 5 degrees [42], and sagittal-plane knee angles during drop jumps with less than 10% error [43].

In the current study, we examined the accuracy of Kinovea for estimating the kinematics of falls from planar video with a resolution (640 x 480 pixels) and frame rate (30 Hz) typical of that acquired by surveillance video cameras. We addressed four objectives. First, we sought to determine the accuracy of Kinovea estimates under ideal video recording conditions, involving the camera axis oriented perpendicular to the plane of the fall [44], and calibration of the video (in m/pixel) based on a 2D grid recorded in the plane of the fall. We measured accuracy by comparing estimates from Kinovea to measures from high speed, 3D motion capture of the linear and angular positions and velocities of the head, torso, pelvis, upper limbs, and lower limbs. Second, we sought to determine how the accuracy of Kinovea was affected by the direction of the fall, and confirm whether our results support previous observations of good accuracy in kinematic estimates from planar video for backward falls [40], and higher errors for sideways falls, due to out-of-plane movements of the body segments [41]. Third, we examined how the accuracy of Kinovea is affected by the camera angle and frame rate (simulated through low-pass filtering the position data), which may vary widely in real-life conditions [35]. Fourth, we examined the accuracy of a simple 1D calibration approach based on the height of the faller, as a practical alternative to a 2D calibration grid in the plane of the fall. Our results provide a basis for understanding what can be accurately measured, and for recommended approaches for using Kinovea to analyze the kinematics of falls from 2D planar video.

## 2. Methods

### 2.1 Participants

**Fig 1:**
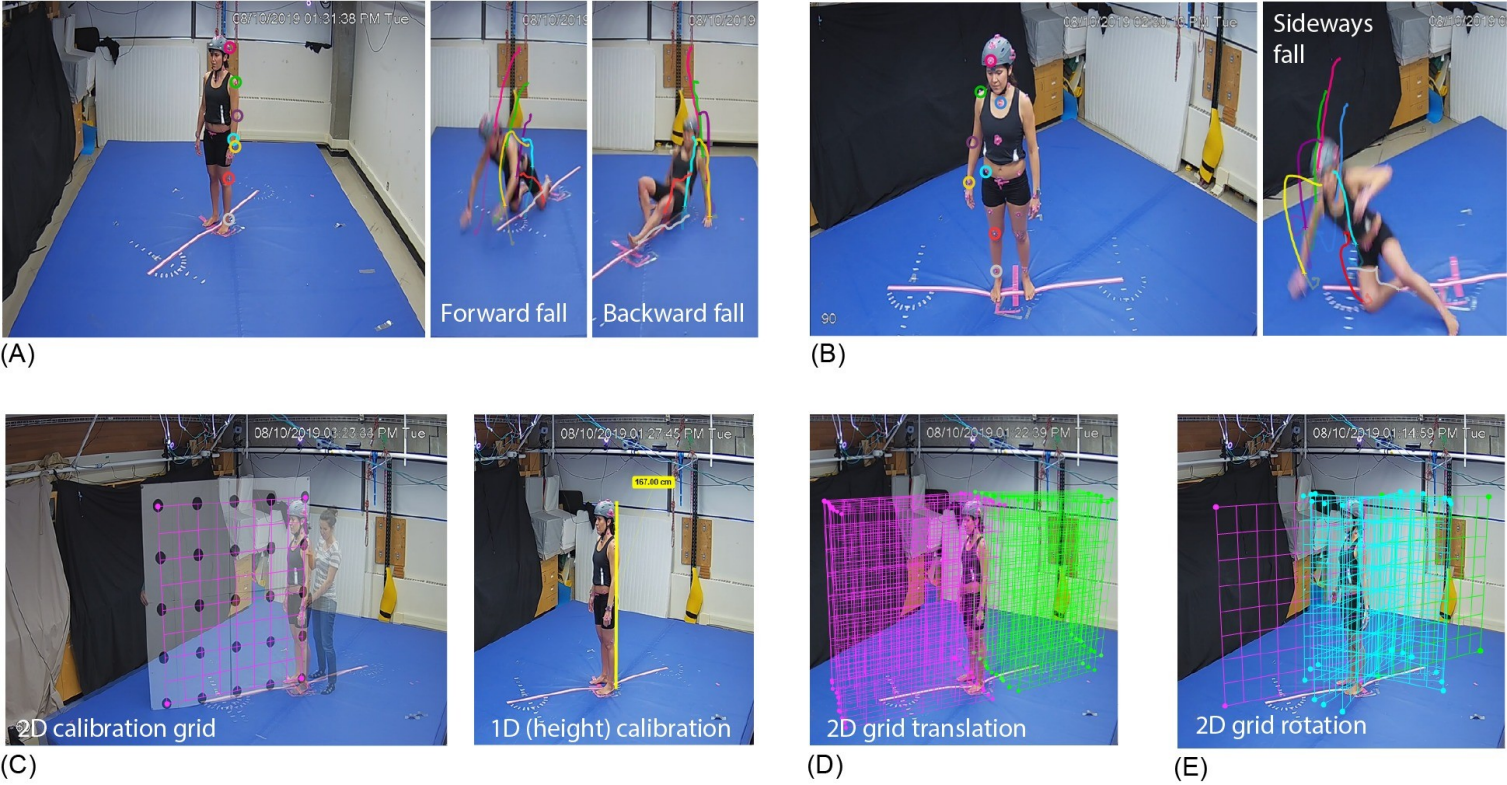
Experimental setup. Experimental setup for (A) forward and backward and (B) sideways falls illustrating Kinovea traces from digitization of key anatomical locations. Right panels display digitized traces from Kinovea of the time-varying positions during the falls of the head (pink), shoulder (green), elbow (purple), wrist (yellow), hip (light blue), knee (red), ankle (grey) and sternum (dark blue). Pink tape of the mat displays the plane of the fall. We examined how the accuracy of kinematic outcomes from Kinovea depended on (C) calibration technique (2D calibration grid versus 1D calibration based on participant height), (D) calibration frame translation (purple grids for forward falls and green grids for backward falls), (E) calibration grid rotation (blue grids), and camera view with respect to the fall (30, 60 and 90 degree views shown in (A), (C), and (B), respectively).

Thirty six falls were performed by three young adults who participated in this study (two women aged 32 and 22 years, and one man aged 34 years). None of the participants had a recent history of muscle strain, joint sprain, bone fracture, or concussion. The study was approved by the Office of Research Ethics at Simon Fraser University, and participants provided written informed consent prior to participating. Furthermore, all individuals whose image is shown in Fig 1 have given written informed consent (as outlined in PLOS consent form) to publish these images.

### 2.2 Protocol for falling experiments

Our protocol was designed to evaluate the accuracy of Kinovea for various fall directions and causes of imbalance. During the experiment, participants stood on a 4.4 x 3.8 m custom-built platform (Fig 1) that could rapidly translate in different directions in the horizontal plane via linear motors (Trilogy System Corporation, Webster, Texas, USA). The surface of the platform was covered by gym mats (30 cm thick) to prevent injuries during falls.

One set of trials was designed to simulate falls due to slipping or tripping. In these trials, falls were induced by suddenly translating the platform forward, backward, or sideways. The perturbation involved acceleration at 10 m/s^2^ to a velocity of 2.2 m/s, followed by a period of constant velocity, and finally deceleration, over a total duration of 0.6 s [29]. A second set of trials was designed to simulate falls due to incorrect weight shifting (e.g., often characterized by excessive sway) [35]. In these trials, falls were self-initiated by the participant. In all trials, participants were instructed to “fall naturally,” and wore a securely fitted helmet (Aspect, Smith (Sun Valley, USA)). Our analysis includes a total of 36 falls (six perturbation-based and six self-initiated falls in each of the forward, backward, and sideways directions).

### 2.3 Raw kinematic data from video (Kinovea) and motion capture (Qualisys)

During each trial, body movements were recorded for Kinovea analysis with four planar video cameras (Lorex LNZ44P4B, Markham, Canada), at a frame rate of 30 Hz and resolution of 640 x 480 pixels, selected to match common features of video from security cameras and sporting events. The Lorex cameras were positioned with their axes perpendicular to the plane of the fall (anterior and posterior to the participant during sideways falls, and to the left and right during forward and backward falls), and at angles of 30 and 60 degree to the plane of the fall (Fig 1). Using Kinovea (version 0.8.27), we manually digitized video footage from each camera angle frame-by-frame to determine vertical and horizontal marker positions from 10 frames before initiation of the fall to 10 frames after landing from the fall (cessation of movement). We refer to corresponding kinematic outcomes as “Kinovea” data.

We also recorded the time-varying, 3D positions of body segments with an 8-camera, passive-marker motion capture system at 600 Hz (Qualisys MIQUS, Gothenburg, Sweden). Each camera had a resolution of 6 megapixels, and the system was calibrated before each session. We refer to kinematic data from 3D motion capture as “Qualisys.” For forward and backward falls reflective markers were placed (Fig 1A and 1B) on the participants’ head (lateral aspects of helmet), shoulders (lateral to the glenohumeral joint), lateral humeral epicondyles, radial styloid processes, greater trochanters (GT), lateral femoral condyles, and lateral malleoli. For sideways falls, reflective markers were placed on participants’ head (front of helmet), manubrium, acromion processes, lateral humeral epicondyles, radial styloid processes, anterior superior iliac spines (ASIS), midpoint of femoral condyles, and midpoint of malleoli. Pink tape was positioned around the markers to increase marker visibility in the videos.

Raw unfiltered position data from both Kinovea and Qualisys are provided as supporting information (S2–S5 Tables).

### 2.4 Data analysis

#### 2.4.1 Identification of Qualisys “ground truth” signals

**Fig 2:**
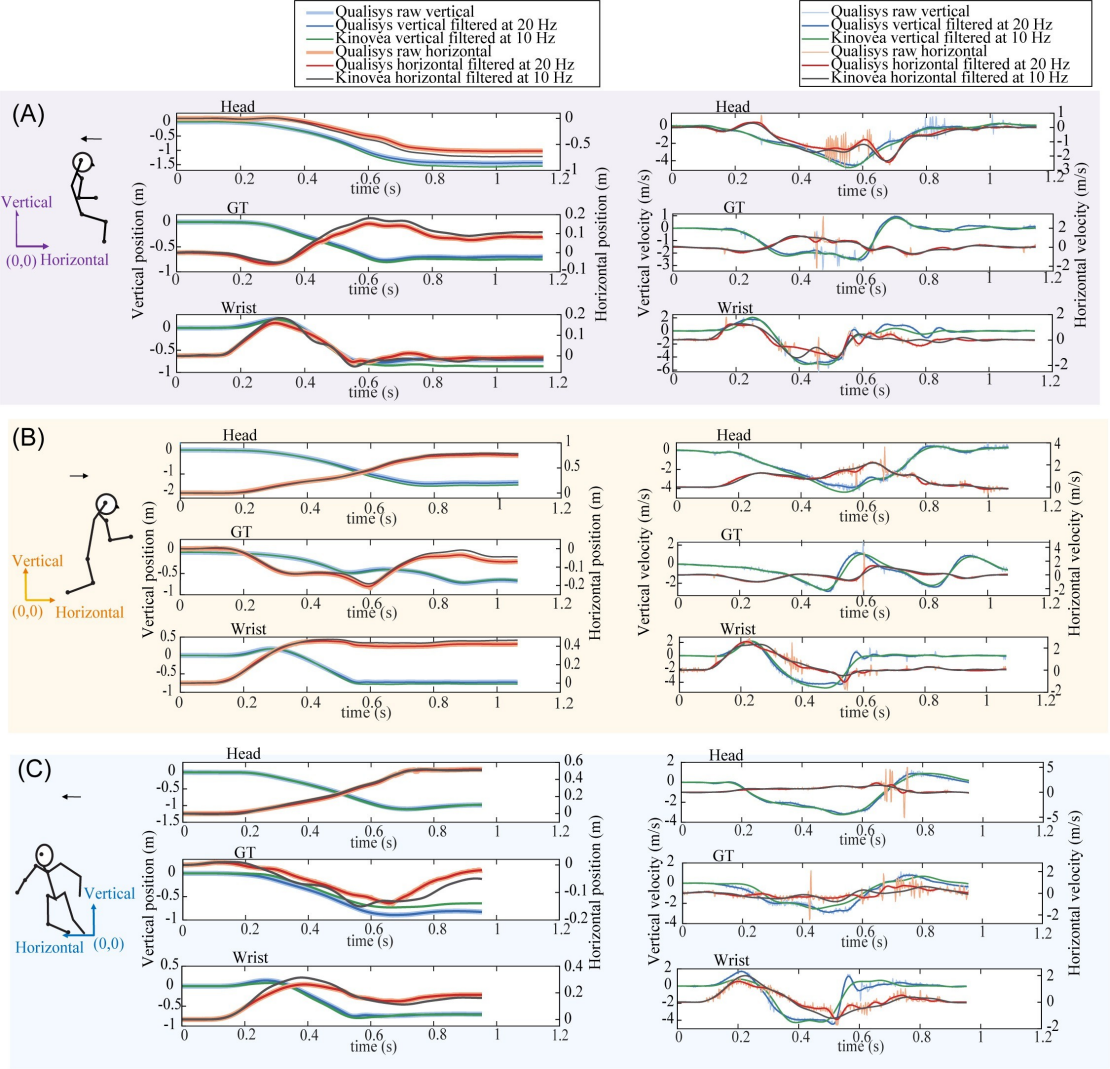
Time-varying linear positions and velocities measured from 3D motion capture (Qualisys) and Kinovea. The images show perturbation-based falls experienced by a 32 year old woman in the (A) backward fall direction, (B) forward fall direction, and (C) sideways fall direction. All traces are between onset of platform movement (time = 0) and end of fall (cessation of movement). Raw unfiltered data show considerable non-physiological noise. A 20 Hz low pass filter of Qualisys position data retained 96% of the energy content for 96.7% of the vertical velocity traces.

To arrive at a “ground truth” set of kinematic data from Qualisys (for comparison with other signals), we identified cut-off frequencies for low-pass filtering of Qualisys position data that removed non-physiological high frequency noise. From Fourier analysis, we found that less than 4% of the signal energy content was above 20 Hz, for 96.7% of all unfiltered vertical velocity traces (n = 365). Accordingly, Qualisys ground truth signals were based on position data processed with a 4th order, dual-pass Butterworth low-pass filter using a 20 Hz cut-off frequency. Visual inspection confirmed that the 20 Hz low-pass filter preserved the physiological components of the movement (Fig 2A–2C).

#### 2.4.2 Processing of kinematic data

Kinovea data were converted from pixels to mm using the built-in calibration features of Kinovea software. The data were then low-pass filtered with a dual-pass 4th-order Butterworth filter, upsampled to 600 Hz, and temporally synchronized with Qualisys ground truth signals based on cross-correlation of head vertical velocity signals. We systematically examined how the accuracy of Kinovea signals depended on the filter cut-off frequency, and on the technique used in calibrating the videos (see section 2.4.5 below).

For both Qualisys and Kinovea, initial values were subtracted from horizontal (X axis) and vertical (Z axis) position data, to account for differences in the origins of the coordinate systems. Absolute angles of body segments were calculated from position data using the inverse tangent function. We focused on segment angles; rather than relative (joint) angles, since falls researchers are often interested in the orientation of body segments with respect to the vertical or the ground [45,46]. Head angles were calculated from head and shoulder markers during forward and backward falls, and head and sternum markers during sideways falls. Angles were calculated for the torso from shoulder and GT or ASIS, for the thigh from GT or ASIS and knee, for the upper arm from elbow and shoulder, for the forearm from elbow and wrist, and for the shank from knee and ankle markers. Linear and angular velocities were determined from numerical differentiation of position data using a first order central difference approach. Peak velocities (between fall initiation and end of fall) were identified for each anatomical location of interest.

#### 2.4.3 Accuracy measures

We measured signal accuracy by comparing Qualisys ground truth data to either data derived from Kinovea, or data derived from alternative processing of Qualisys data (see section 2.4.6 below). Our measures of accuracy were: (a) the Root Mean Square Error (RMSE) in signals between the onset and end of the fall; (b) the RMSE normalized to the signal amplitude (NRMSE); and (c) the raw and absolute differences in peak velocities.

#### 2.4.4 Inter-rater reliability of Kinovea data

We examined the inter-rater reliability of kinematic signals from Kinovea by comparing results from two raters, who independently digitized and calibrated videos of whole-body movements from three perturbation-based falls. RMSEs in linear position and velocity throughout the fall were compared for all camera angles, along with Intraclass Correlation Coefficients (ICC) (SPSS for Windows, version 25, IBM Corp., Armonk, N.Y., USA).

The mean RMSE between two raters was 0.011 m (SE = 0.001) for position, or 2.4% (SE = 0.2) of the signal amplitude, and 0.13 m/s (SE = 0.01) for velocity, or 4.8% (SE = 0.3) of the signal amplitude. ICC values and 95^th^ confidence intervals were 1.00 (1.00–1.00) for linear position and 0.985 (0.984–0.986) for linear velocity.

#### 2.4.5 Effect of cut-off frequency and calibration technique on accuracy of Kinovea data

We examined the accuracy of Kinovea data for cut-off frequencies of 3, 5, 7, 10, 12, and 14 Hz for low-pass filtering of position data. 14 Hz was selected as the maximum cut-off frequency for filtering the 30 Hz data, based on Nyquist considerations. We also examined how the accuracy of Kinovea outcomes differed between two approaches available in Kinovea for calibrating the videos (i.e., converting from pixels to m): a 2D grid versus a 1D vertical line (Fig 1C). The 2D grid was of dimensions 1.6 x 1.6 m, and contained a 5 x 5 array of dots spaced 40 cm apart. At baseline, the grid was located in the plane of the fall. The 1D vertical line was placed in a frame when the participant was standing prior to the onset of the fall. The line extended from the base of the foot to the top of the head, and was set equal to participant height. We also examined how signal accuracy was affected by: (a) translating the grid by ±10, ±20, ±30, ±40, and ±50 cm from the plane of the fall, where negative and positive values reflect translation away from and towards the camera, respectively (Fig 1D); (b) rotating the grid ±15, ±30, and ±45 degrees from the plane of the fall (Fig 1E); and (c) introducing errors in participant height of ±10 cm from the actual values.

#### 2.4.6 Qualysis “lower bound” signals

We examined the bandwidth required for accurately capturing body movements during falls by comparing Qualisys ground truth data to Qualisys signals low pass filtered at lower cut-off frequencies. When compared to ground truth data, Qualisys signals filtered at 10 Hz had NRMSE less than 3% for horizontal and vertical position and velocity. Errors increased to 4% at 7 Hz, 5% at 5 Hz, and 8% at 3 Hz (Table 1). Peak vertical velocities matched ground truth values to within 0.06 m/s (3%) at 10 Hz, 0.13 m/s (5%) at 7 Hz, 0.22 m/s (9%) at 5 Hz, and 0.45 m/s (17%) at 3 Hz (Table 1 and Fig 2). Based on these findings, we regarded 10 Hz as an appropriate “lower bound” filter cut-off frequency that provides over 95% accuracy in capturing body segment positions and velocities during falls, and represents the theoretical upper limit on the accuracy of video cameras capturing at 10 Hz.

**Table 1:** Errors between position and velocity signals from Qualisys and Kinovea from different cut-off frequencies.

	Vertical position		Horizontal position		Vertical velocity				Horizontal velocity			
	RMSE (m)	NRMSE (%)	RMSE (m)	NRMSE (%)	RMSE (m/s)	NRMSE (%)	Raw difference in peak velocity (m/s)	Percent difference in peak velocity (%)	RMSE (m/s)	NRMSE (%)	Raw difference in peak velocity (m/s)	Percent difference in peak velocity (%)
Q20-K14	0.051±0.002	9.2±0.6	0.035±0.002	6.0±0.3	0.22±0.01	6.5±0.2	-0.04±0.02	-1.3±0.9	0.16±0.01	7.3±0.2	0.03±0.02	0.9±1.0
Q20-K12	0.051±0.002	9.2±0.6	0.035±0.002	6.0±0.3	0.22±0.01	6.5±0.2	-0.04±0.02	-1.1±0.9	0.16±0.01	7.2±0.2	0.32±0.02	1.2±1.0
Q20-K10	0.050±0.002	9.2±0.6	0.035±0.002	6.0±0.3	0.22±0.01	6.5±0.2	-0.02±0.02	-0.5±0.9	0.16±0.01	7.2±0.2	0.05±0.02	2.1±1.0
Q20-K7	0.050±0.002	9.2±0.6	0.035±0.002	6.0±0.3	0.23±0.01	6.7±0.2	0.03±0.02	1.2±0.9	0.16±0.01	7.2±0.2	0.10±0.02	5.0±1.0
Q20-K5	0.051±0.002	9.2±0.6	0.035±0.002	6.0±0.3	0.25±0.01	7.2±0.2	0.11±0.03	4.3±1.0	0.17±0.01	7.7±0.2	0.18±0.02	9.6±0.9
Q20-K3	0.051±0.002	9.4±0.6	0.037±0.001	6.4±0.3	0.32±0.01	8.9±0.2	0.33±0.03	12.0±1.2	0.22±0.01	9.8±0.2	0.37±0.02	19.6±1.0
Q20-Q14	0.001±0.001	0.1±0.1	0.001±0.001	0.1±0.1	0.03±0.01	0.7±0.1	0.02±0.01	0.8±0.1	0.02±0.01	1.0±0.1	0.02±0.01	1.1±0.1
Q20-Q12	0.001±0.001	0.1±0.1	0.001±0.001	0.1±0.1	0.04±0.01	1.1±0.1	0.03±0.01	1.3±0.1	0.03±0.01	1.5±0.1	0.03±0.01	1.8±0.0
Q20-Q10	0.001±0.001	0.2±0.1	0.001±0.001	0.1±0.1	0.06±0.01	1.6±0.1	0.06±0.01	2.3±0.2	0.05±0.01	2.1±0.1	0.06±0.01	3.0±0.2
Q20-Q7	0.002±0.001	0.4±0.1	0.001±0.001	0.3±0.1	0.11±0.01	2.8±0.1	0.13±0.01	4.8±0.4	0.08±0.01	3.3±0.1	0.12±0.01	6.2±0.4
Q20-Q5	0.004±0.001	0.8±0.1	0.003±0.001	0.6±0.1	0.16±0.01	4.3±0.1	0.22±0.01	8.3±0.5	0.11±0.01	4.8±0.1	0.21±0.01	10.7±0.5
Q20-Q3	0.010±0.001	2.0±0.1	0.007±0.001	1.7±0.10	0.27±0.01	7.2±0.1	0.45±0.02	16.6±0.8	0.18±0.01	7.7±0.1	0.41±0.02	21.0±0.7

**NOTES:** Comparisons are between Qualisys ground truth signals (Q20) and either Kinovea signals (top six rows) or Qualisys signals (bottom six rows) from low-pass filtering of position data at different cut-off frequencies (e.g., 14 Hz for “K14” and “Q14”). Results show mean ± 1 SE values averaged across all body parts and fall directions. Results are specific to Kinovea data from the 90 degree camera angle, calibrated with the calibration frame located in the plane of the fall. For positions, values less than 0.001 m are rounded up. For velocities, values less than 0.01 m/s are rounded up. Percent differences (NRMSE and peak velocities) are calculated for each fall and then averaged across falls.

#### 2.4.7 Magnitude of out-of-plane movement during falls

Finally, we analyzed Qualisys data to quantify peak displacements and velocities of body segments in the “out-of-plane” (Y) axis during falls, which could not be captured by our 2D Kinovea analysis. We also compared horizontal X positions and velocities to resultant XY signals from Qualysis.

### 2.5 Statistical analysis

We used one-way ANOVAs to examine the effect of the following methodological variables on the accuracy of Kinovea signals: Kinovea filter cut-off frequency, camera angle, fall direction, body part, and calibration technique. Each fall was treated as an independent measure. Fall direction was analyzed with independent samples ANOVAs because the comparisons were between different falls. All other variables were analyzed with repeated measures ANOVAs (with the fall treated as a random factor) because these variables related to different approaches (or treatments) for collecting or processing data from the same set of falls. In case of significant main effects, Tukey’s Post hoc analysis was used to test pair-wise comparisons. All ANOVA analyses were conducted using JMP software (Version 15. SAS Institute Inc., Cary, NC) using a significance level of α<0.05.

## 3. Results

**Fig 3:**
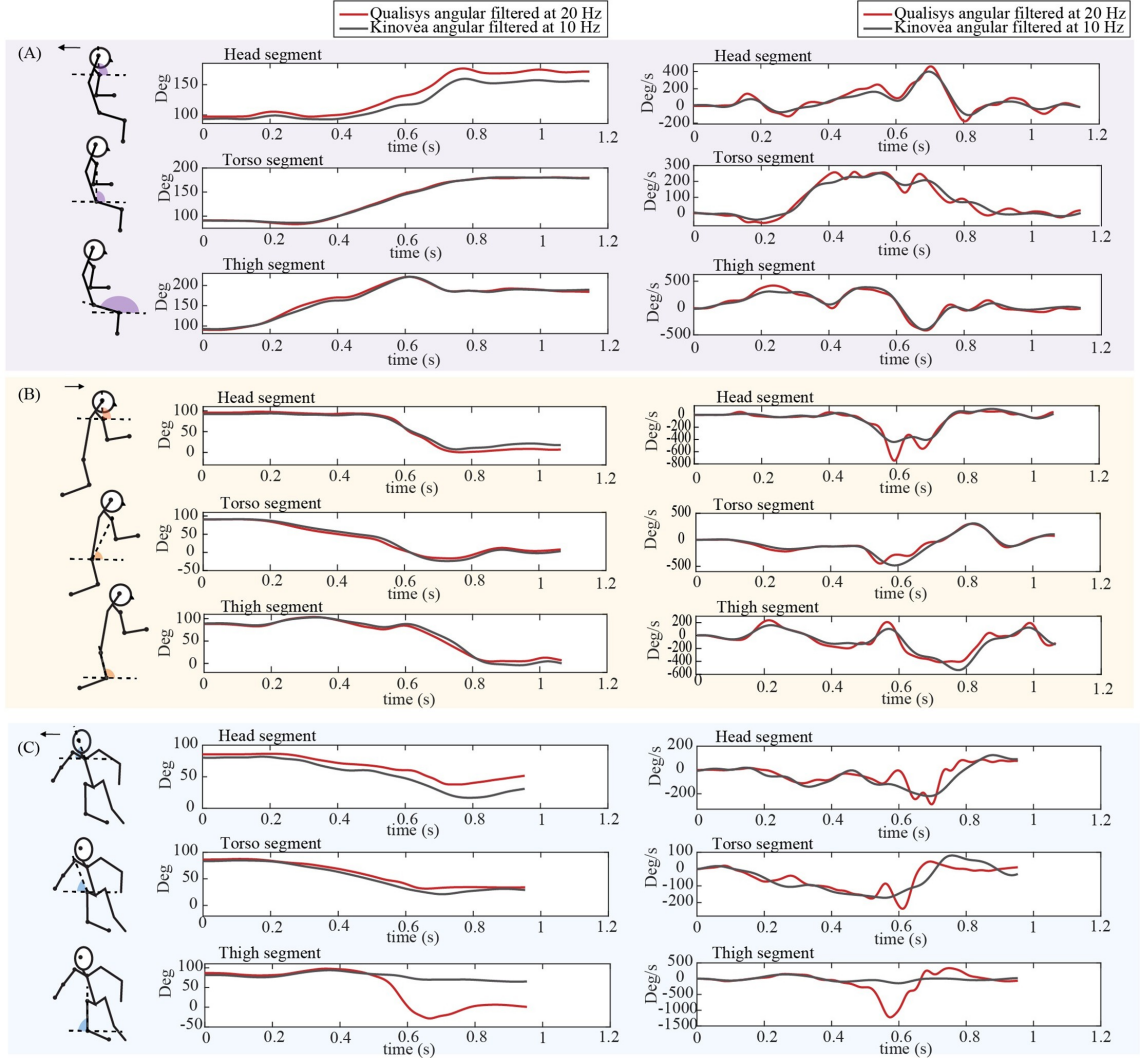
Angular positions and velocities measured from 3D motion capture (Qualisys) and Kinovea. The traces show time-varying angular positions (with respect to the horizontal) and velocities for selected body segments, measured for (A) backward, (B) forward, and (C) sideways perturbation-based falls. Data are for the same falls as shown in Fig 2.

Based on Qualisys ground truth signals, the head displaced by average vertical and horizontal distances of 1.109 and 0.976 m, rotated by 50.9 degrees, and experienced peak vertical and horizontal velocities of 3.04 and 2.26 m/s and peak angular velocities of 415 degree/s. Figs 2 and 3 show time-varying linear and angular positions and velocities for a sample backward, forward, and sideways fall.

Unless specified otherwise, the comparisons below are for Kinovea results from cameras oriented at 90 degree (camera in front during sideways falls and to the right during forward and backwards falls), the calibration grid placed in the plane of the fall, and low-pass filtering of position data with a 10 Hz cut-off frequency.

### 3.1 Effect of filtering on the accuracy of fall kinematics measured by Kinovea

**Fig 4:**
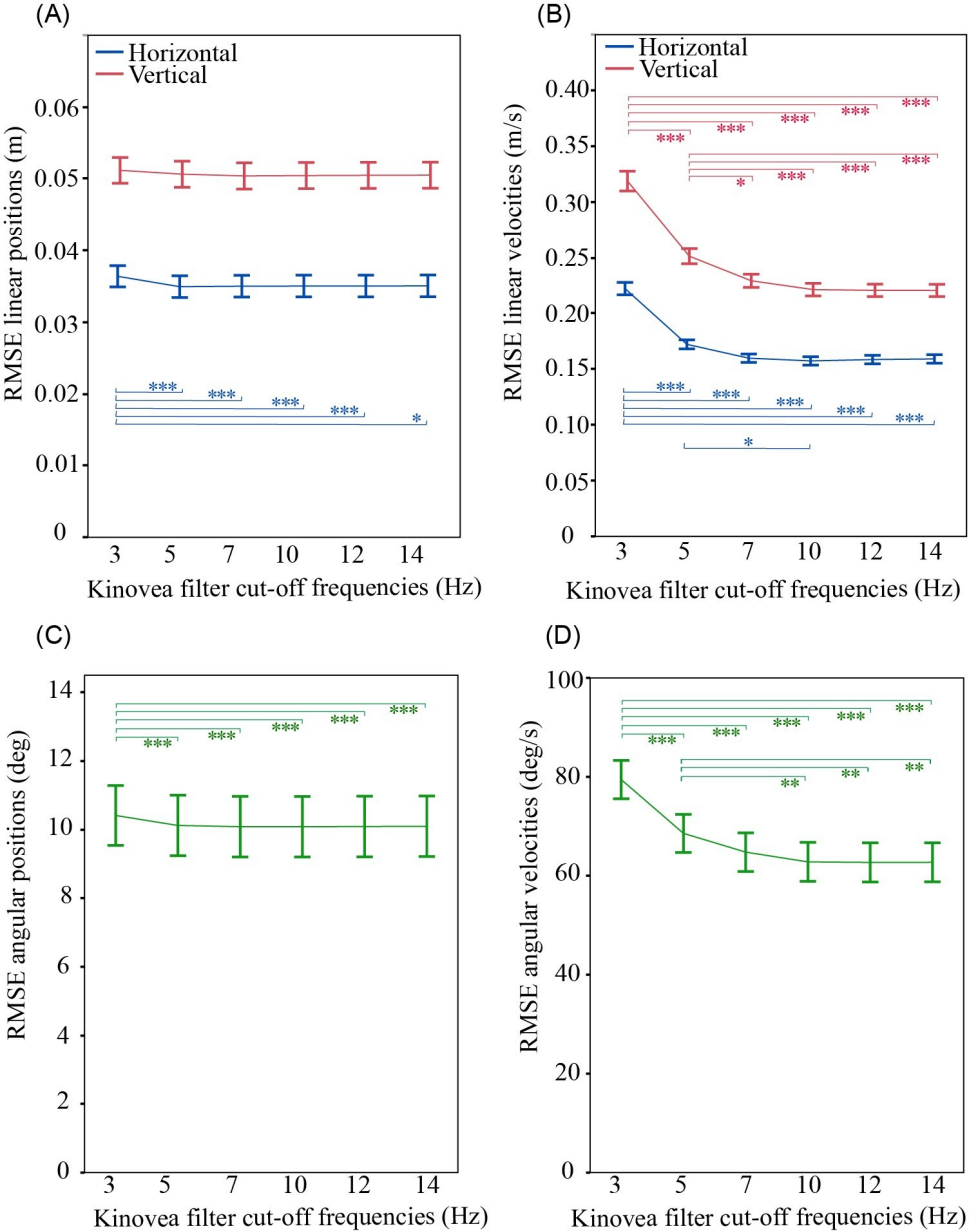
Effect of low-pass filter cut-off frequency on the accuracy of Kinovea estimates. (A) Linear position, (B) linear velocity, (C) angular position, and (D) angular velocity. The plots show root mean square errors (RMSE) for Kinovea data filtered at cut-off frequencies of 3, 5, 7, 10, 12 and 14 Hz, based on comparison with Qualisys ground truth signals. Results are specific to Kinovea data from the 90 deg camera angle, calibrated with the calibration frame located in the plane of the fall. Data points show mean values averaged across all body parts and fall directions with error bars showing ± 1 SE. * p≤0.05, ** p≤0.01, *** p≤0.001.

Based on comparison to Qualisys ground truth signals, the accuracy of Kinovea signals depended on the cut-off frequency used in low-pass filtering Kinovea position data (Table 1 and Fig 4). There was a significant effect of cut-off frequency on mean RMSEs for horizontal (but not vertical) and angular position (p≤0.0002), and for vertical, horizontal, and angular velocity (p<0.0001). Post hoc analysis showed that RSMEs for all outcomes were not different between 7, 10, 12, and 14 Hz. RSMEs for position were lower at 3 Hz (p≤0.0120), and for velocities at 5 Hz (p≤0.0299), than for higher cut-off frequencies. With a 10 Hz cut-off frequency, mean RMSEs were 0.050 m (SE = 0.002) or 9.2% of the total signal amplitude for vertical position, 0.036 m (SE = 0.002) or 6% for horizontal position, 0.22 m/s (SE = 0.01) or 6.5% for vertical velocity, 0.16 m/s (SE = 0.01) or 7.2% for horizontal velocity, 10.1 degree (SE = 0.9) or 13.0% for angular position, and 63 degree/s (SE = 4) or 10.0% for angular velocity (Table 1, Fig 4 and S1 Table).

### 3.2 Effect of fall direction and body part on the accuracy of fall kinematics measured by Kinovea

**Fig 5:**
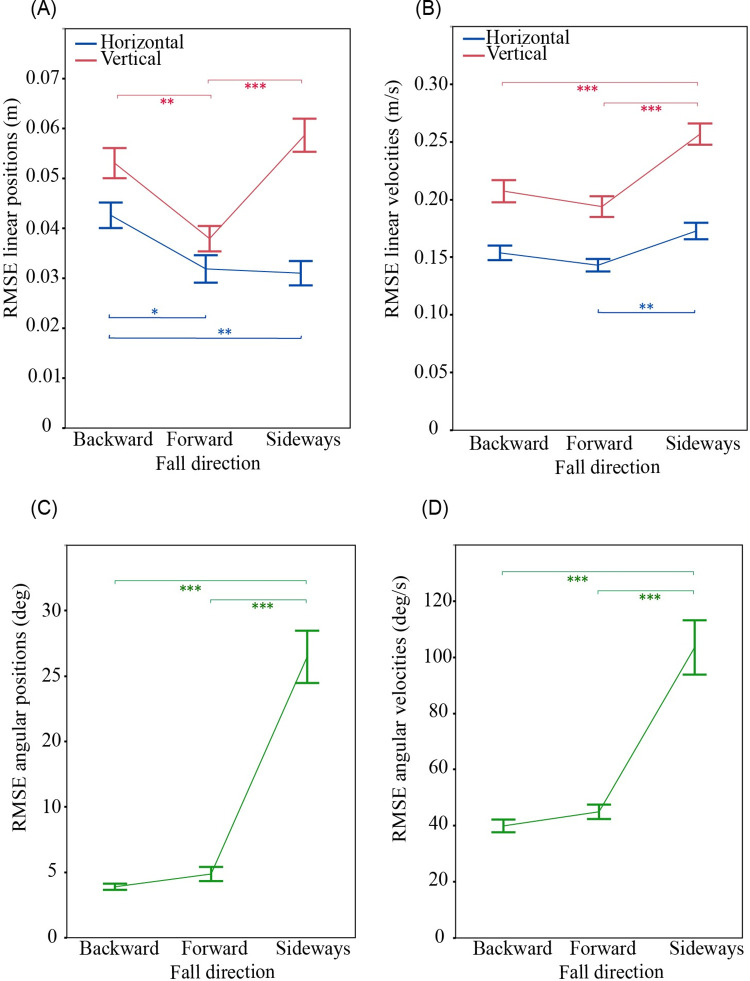
Effect of fall direction on accuracy of Kinovea estimates. (A) Linear position, (B) linear velocity, (C) angular position, and (D) angular velocity. RMSEs are based on comparison of Qualisys ground truth signal to Kinovea data from the 90 deg camera angle, calibrated with the calibration frame located in the plane of the fall, with position data filtered at 10 Hz. Data points show mean values averaged across all body parts with error bars showing ± 1 SE. * p≤0.05, ** p≤0.01, *** p≤0.001.

There was a significant effect of fall direction on the accuracy of Kinovea for all linear and angular measures (p≤0.0043; Fig 5). Post hoc analysis showed that all outcomes except horizontal positions had smaller RMSEs in forward than sideways falls (p≤0.0035). The differences were greatest for angular positions and velocities, where NRMSEs averaged 25.6 and 15.6% in sideways falls, and 6.7 and 7.2% for forward and backward falls combined. Angular positions, and vertical and angular velocities, were more accurate in backward than sideways falls (p≤0.0005). Linear positions but not velocities were less accurate in backward than forward falls (p≤0.0122).

**Fig 6:**
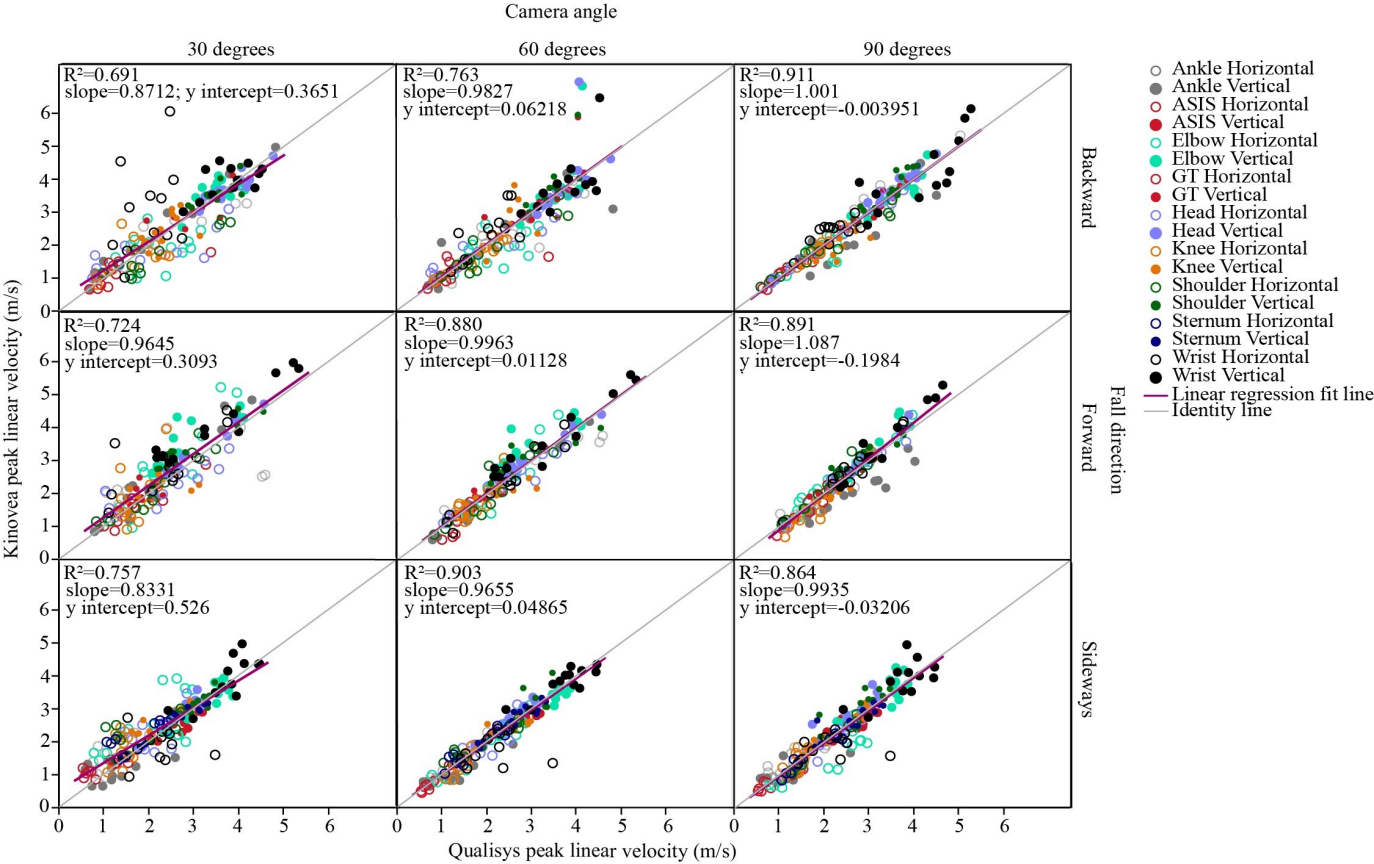
Effects of camera angle and fall direction on agreement between Kinovea and Qualisys peak velocities. The graphs compare peak vertical and horizontal velocities for various anatomical locations. Results are specific to the calibration grid located in the plane of the fall and Kinovea velocities differentiated from position data filtered with a cut-off frequency of 10 Hz.

For the 90 degree camera, R^2^ values for the correlations between peak vertical velocities from Kinovea and Qualisys were 0.911 for backward falls, 0.891 for forward falls, and 0.864 for sideways falls (Fig 6). The slope of the lines varied between 0.83 and 1.09. The mean signed error averaged 2% or lower for both peak vertical velocity and peak horizontal velocity for the 90 degree camera, and was different than zero only for forward and sideways falls from the 30 degree camera, reflecting no bias in Kinovea estimates from the 60 and 90 degree cameras.

### 3.3 Effect of body part on the accuracy of fall kinematics measured by Kinovea

**Fig 7:**
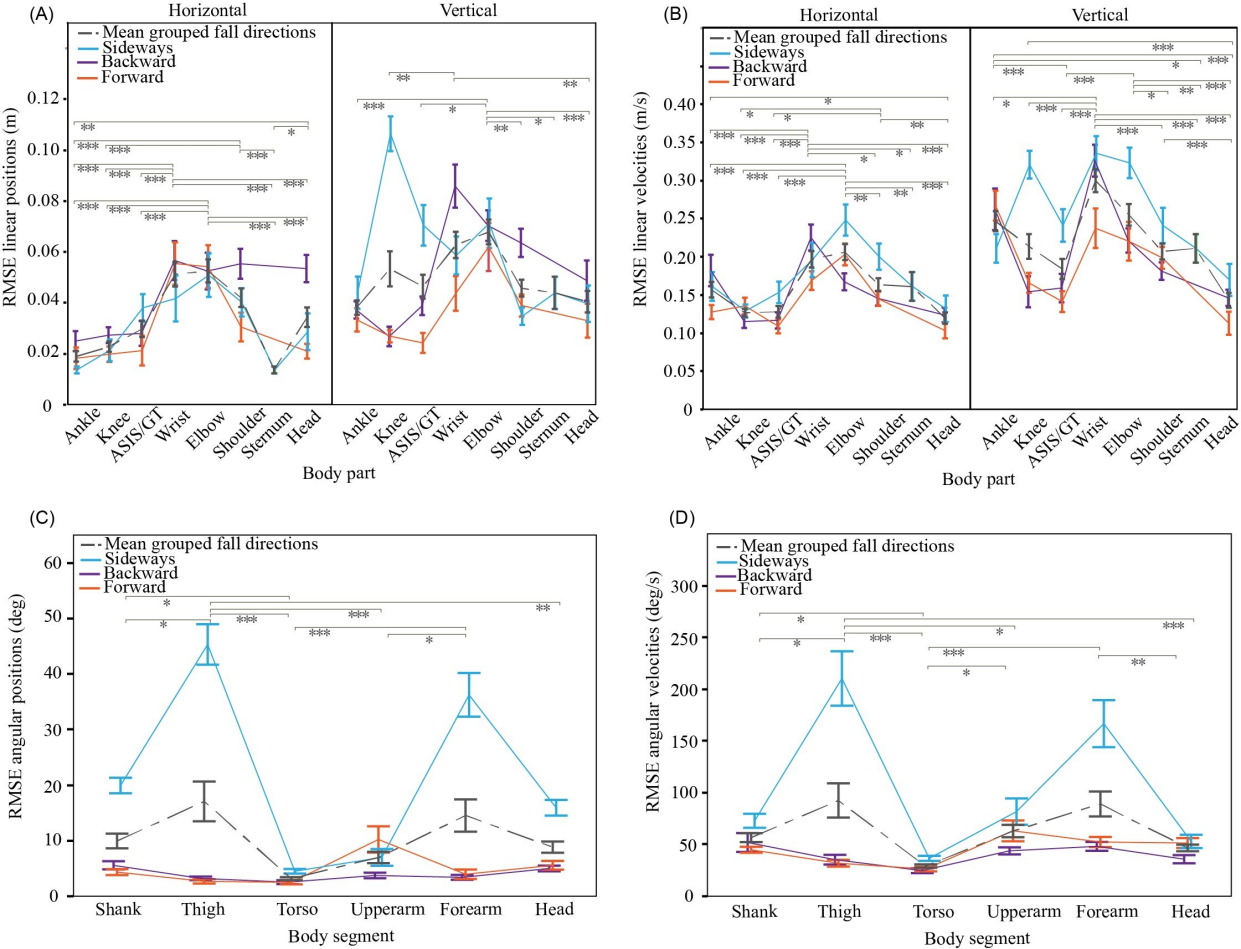
Differences across body sites and fall directions in the accuracy of Kinovea estimates. (A) Linear position, (B) linear velocity, (C) angular position, and (D) angular velocity. Root mean square errors (RMSE) are from comparison between Qualisys ground truth signals and Kinovea estimates based on the 90 deg camera angle, the calibration frame located in the plane of the fall, and filtering of position data with a 10 Hz cut-off frequency. Data points show mean values, and error bars show ± 1 SE (sideways = blue, backward = purple, forward = orange; grey = mean values across all fall directions). Significant differences are shown for mean values across all fall directions (* p≤0.05, ** p≤0.01, *** p≤0.001).

Body parts had a significant effect on mean RMSE for all outcome measures (p<0.0001; Fig 7). For linear measures, the elbow and wrist had at least 20% higher mean RMSEs than other body parts (except for vertical velocities, where the RMSE for ankle was similarly high). For angular measures, the thigh and forearm had at least 42% higher mean RMSEs than other body segments, driven by large errors in sideways falls.

### 3.4 Effect of camera angle on the accuracy of fall kinematics measured by Kinovea

**Fig 8:**
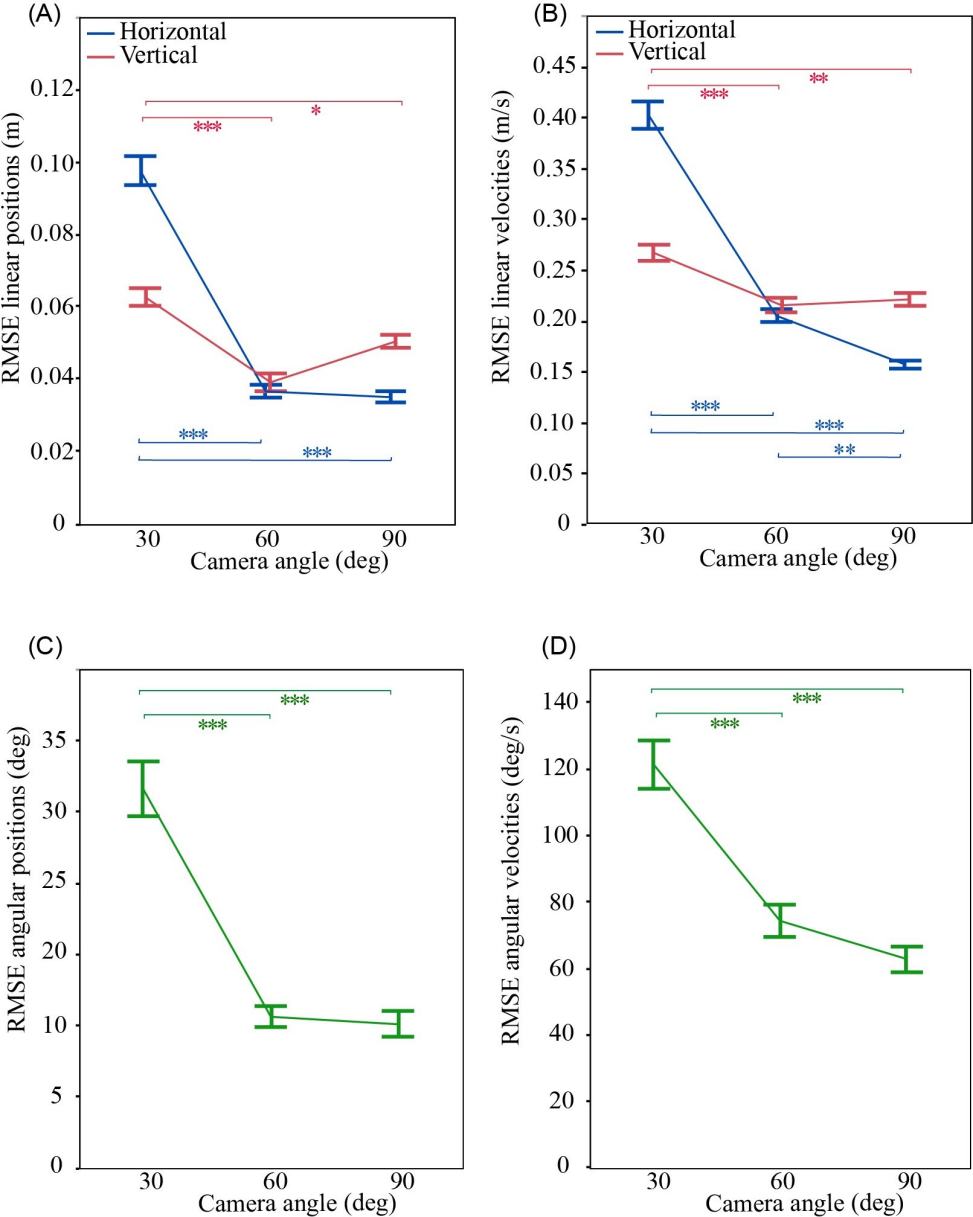
Effect of camera angle on the accuracy of Kinovea estimates. (A) Linear position, (B) linear velocity, (C) angular position, and (D) angular velocity. Root mean square errors (RMSE) are from comparison between Qualisys ground truth signals and Kinovea estimates based on the calibration frame located in the plane of the fall, and filtering of position data with a 10 Hz cut-off frequency. Data points show mean values, and error bars show ± 1 SE. * p≤0.05, ** p≤0.01, *** p≤0.001.

There was a significant effect of camera angle on mean RMSE for all kinematic outcomes (p≤0.0004; Figs 6 and 8). Post hoc analyses showed significantly higher RMSEs for the 30 degree than 90 and 60 degree camera angles for all outcomes (p≤0.0288). At 30 degree, the RMSE values for horizontal and angular measures were over two-fold greater than for 90 degree. There were differences only for horizontal velocities (p = 0.0076) in RMSEs between 60 than 90 degree camera angles.

### 3.5 Effect of calibration on the accuracy of fall kinematics measured by Kinovea

**Fig 9:**
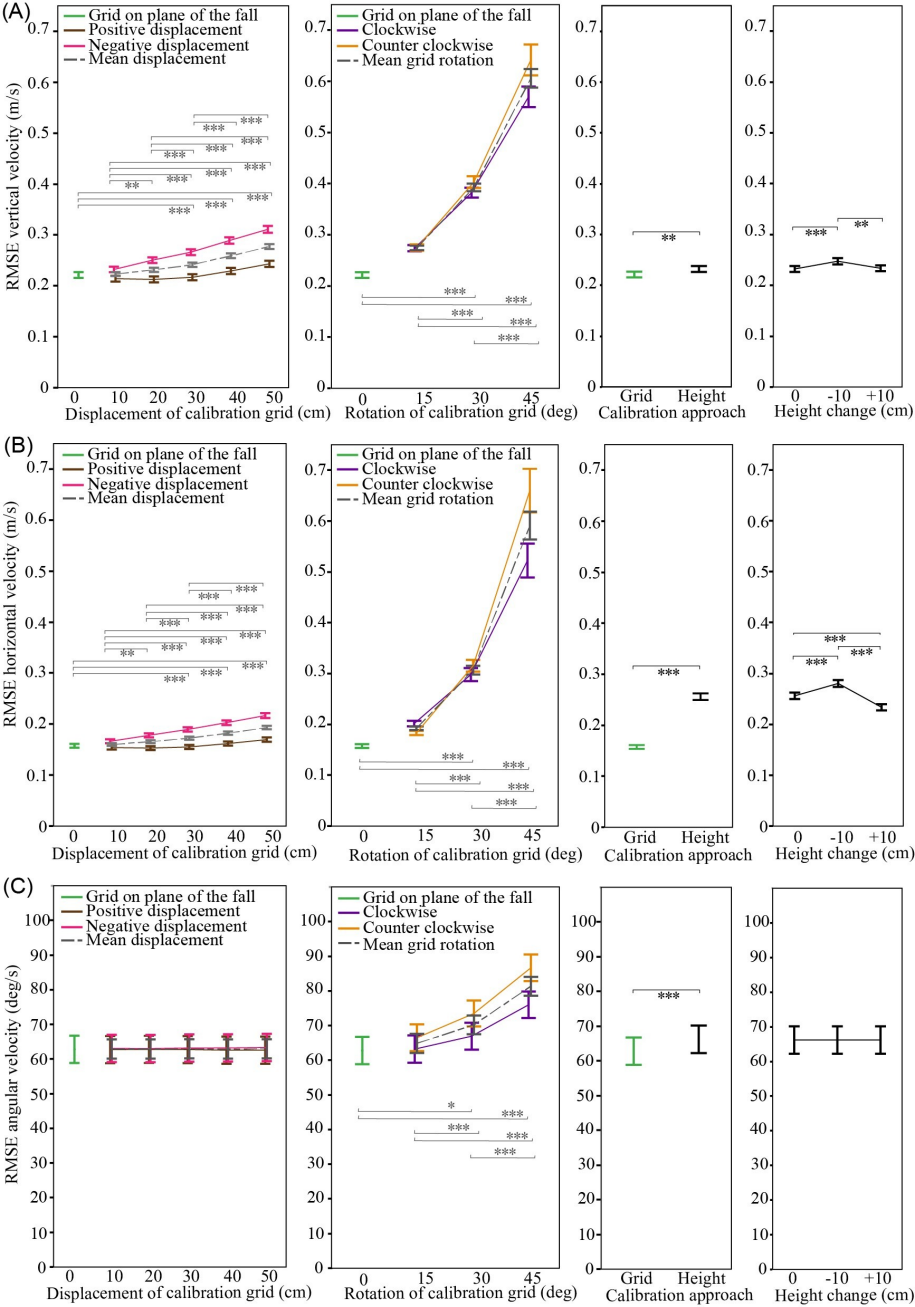
Effect of calibration technique on the accuracy of Kinovea estimates. (A) Vertical velocity, (B) horizontal velocity, and (C) angular velocity. Root mean square errors (RMSE) are from comparison between Qualisys ground truth signals and Kinovea estimates from the 90 deg camera angle, and filtering of position data with a 10 Hz cut-off frequency. Data points show mean values, and error bars show ± 1 SE. Statistical comparisons in two left columns are based on mean values. * p≤0.05, ** p≤0.01, *** p≤0.001.

The accuracy of Kinovea outcomes depended on the orientation of the calibration grid (Fig 9). Both the direction and magnitude of grid translation associated with errors in linear positions and velocities (p<0.0001). Grid translation, averaged for negative and positive displacements (movement of the grid away and towards the camera, respectively), of 10 and 20 cm did not affect the accuracy (p≥0.0922). Average RMSE values for 30 and 50 cm translation were 10 and 24% greater than at baseline. For larger translations, mean RMSEs for linear outcomes increased more for negative than positive translations (p<0.0001). Furthermore, for grid translations in the positive direction there was no effect on accuracy of vertical and horizontal linear velocities for translations up to 40 cm (p≥0.0762) and 30 cm (p≥0.8186). Conversely, negative displacement of the grid beyond 10 cm resulted in reduced accuracy (p<0.0001). Distance of grid translation did not significantly effect mean RMSEs for angular positions (p = 0.0794) and velocities (p = 0.6852).

Rotation of the calibration grid also had a significant effect on the accuracy of all Kinovea outcomes (Fig 9). Mean RMSEs did not differ between 0 and 15 degree, except for vertical linear positions (p = 0.0375), but thereafter increased with grid rotation (p≤0.0185). Again, the effect was greater for linear than angular velocities. For linear velocities, RMSEs at 30 and 45 degree were ~2 and ~3-fold larger, respectively, than values for the grid oriented in the plane of the fall.

Mean RMSEs were higher for all outcomes when videos were calibrated based on participant height versus the calibration grid in the plane of the fall (p≤0.0031; Fig 9). The difference was more pronounced for horizontal velocities (where the mean NRMSE increased from 7.2% to 11.2%; S1 Table) than for vertical velocities (6.5% versus 6.8%). Introducing errors in the value of body height used in the video calibration affected the accuracy of linear (p<0.0001), but not angular measures (p≥0.7644; Fig 9).

### 3.6 Magnitude of out-of-plane movement during falls measured by Qualisys

From Qualisys 3D motion capture data, the peak magnitudes of out-of-plane (Y) horizontal displacement and velocity, averaged over all body parts, were 0.089 (SD = 0.060), 0.099 (0.050), and 0.158 (0.066) m, and 0.73 (0.51), 0.79 (0.48), and 0.96 (0.60) m/s, during backward, forward and sideways falls, respectively. In comparison, peak in-plane (X) horizontal displacement and velocity during backward, forward, and sideways falls averaged 0.664 (0.400), 0.810 (0.528), and 0.658 (0.420) m, and 2.04 (0.88), 2.13 (0.84), and 1.74 (0.68) m/s. Mean out-of-plane movements larger than 0.150 m were observed during backward falls only for the wrist (0.161 (0.084) m; mean peak out-of-plane velocity = 1.27 (0.56) m/s), and during sideways falls for the elbow (0.195 (0.061) m; 2.19 (0.60) m/s), ASIS (0.172 (0.051) m; 0.74 (0.18 m/s)), and knee (0.207 (0.026) m; 1.01 (0.47) m/s). Mean RMSEs between X (1D) and resultant XY (2D) horizontal positions and velocities during backward, forward and sideways falls were 0.010 (0.014), 0.009 (0.016), and 0.023 (0.030) m, and 0.10 (0.10), 0.10 (0.09), and 0.24 (0.30) m/s, respectively.

## 4. Discussion

We examined the accuracy of Kinovea software (when compared to high-speed, 3D motion capture) in estimating the kinematics of the body during falls from video footage collected by standard surveillance cameras (30 Hz, 640 x 480 resolution). We also examined how the accuracy of Kinovea depended on fall direction, camera angle, video calibration technique, and cut-off frequency for low-pass filtering of position data. Below, we provide guidance for those considering the use of Kinovea for analyzing the kinematics of video-captured falls. We discuss the accuracy of Kinovea observed under both “ideal” and non-ideal conditions, where ideal conditions involved: (a) the camera axis oriented perpendicular (90 degrees) to the plane of the fall, (b) the video calibrated from a 2D grid recorded in the plane of the fall, and (c) a 10 Hz cut-off frequency used for low-pass filtering of position data. We also highlight the conditions where the accuracy of specific parameters was substantially lower than values observed under ideal conditions, and discus the potential reasons for the observed trends.

We stress that, for a given user, the acceptability of the observed errors will depend on the user’s required level of accuracy, which in turn depends on the study goals and design. Consider, for example, a situation where a user is interested in comparing peak vertical velocities in forward versus backward falls [41], or in falls experienced by women versus men [47]. For these applications, the small error of Kinovea in measuring peak vertical velocity over a wide range of conditions should allow the user to detect small differences between conditions (if they exist). In contrast, if the user instead wishes to compare the time-varying angular velocities of body segments [48], for which Kinovea had a substantially higher measurement error, the (actual) difference between conditions may need to be quite large, to overcome the inherent errors and be detected from Kinovea-based analysis. Supplementary S1 Table allows readers to identify the expected level of accuracy for specific combinations of kinematic outcome, body part, fall direction, and approach for video collection and calibration.

Under ideal conditions, Kinovea estimated vertical and horizontal positions and velocities over the duration of the fall to within 9% error, angular positions and velocities to within 13% error, and peak vertical and horizontal velocities to within 2% error and with no bias (or constant error). These baseline errors were likely due to human inaccuracy in digitization of the video footage with Kinovea, and perhaps the limited (640 x 480) resolution of the video. It is unlikely that the errors arose from systemic causes (since Kinovea was just as likely to overestimate as underestimate peak velocities), or from limitations related to the 30 Hz video frame rate. Our results agree with previous studies which found that the frequency content of body movements during falls is between 1–10 Hz [32,39], and therefore capable of being captured accurately from 30 Hz video. From Qualisys 3D motion capture at 600 Hz, we found that 96% of the energy in kinematic signals was below 20 Hz, and a 10 Hz cut-off frequency for low-pass filtering of position data successfully filtered out non-physiological noise with minimal distortion of the underlying signal, even for rapidly-moving body parts such as the hands. For Kinovea, when compared to 10 Hz, filtering at 3 Hz caused velocity errors to increase 1.4-fold. However, the errors in all Kinovea outcomes did not change over cut-off frequencies between 7 and 14 Hz (the maximum value based on Nyquist considerations). While further research is required, our results suggest that data from 15 Hz cameras, filtered at 7 Hz, may provide a level of accuracy similar to the values we observed.

We found that the accuracy of Kinovea depended on fall direction. Since Kinovea involves a 2D analysis of planar video, its accuracy will be reduced by out-of-plane movements of body segments (orthogonal to the plane of the video image). From our Qualisys 3D measures, out-of-plane movements were largest at the knee and elbow in sideways falls, and at the wrist in backward falls. This explains why, when compared to other fall directions, Kinovea errors in sideways falls were about 4-fold higher for thigh and forearm angular position and velocity, and about 2-fold higher for knee and elbow vertical velocity. Furthermore, errors in wrist vertical velocity were about 2-fold higher in backward and sideways falls, than in forward falls. Users should be cautious in relying on Kinovea to yield accurate results for these particular combinations of fall direction and kinematic outcome.

Errors were more stable across fall directions for kinematics of the head, sternum, and pelvis. In particular, we found encouraging evidence for the accuracy of Kinovea in estimating pelvis impact velocities in sideways falls, which are relevant to the cause and prevention of hip fractures [49,50]. Unlike Choi et al. [41], who observed errors that they deemed as unacceptably high for peak pelvis velocities from digitizing planar video of sideways falls (mean error = 1.01 m/s (SD = 0.59) for vertical velocity, and 0.13 m/s (SD 0.54) for horizontal velocity), we found that Kinovea estimated peak velocities of the ASIS during sideways falls to within an error of 0.12 m/s (3.9%) for vertical velocity, and 0.14 m/s (10.7%) for horizontal velocity. It is difficult to explain why the errors observed by Choi for sideways falls were similar to ours for peak horizontal pelvis velocity, but much higher for peak vertical pelvis velocity, but this may relate to Choi et al. examining only a single fall in each of the forward, sideways and backward directions, while we analyzed 12 falls in each direction.

Since real-life falls may rarely occur in planes that are perpendicular to the axis of a nearby surveillance camera, we explored how the accuracy of Kinovea outcomes differed between 30, 60 and 90 degree camera angles. We found that errors in vertical kinematics were relatively unaffected by camera angle, with the 30 degree camera providing less than 12% error for vertical positions and 8% error for vertical velocities. This suggests that Kinovea is appropriate for estimating vertical kinematics for a wide range of camera angles. However, the camera angle more strongly affected the accuracy of Kinovea estimates of horizontal and angular kinematics, likely due to its effect on the range of observable horizontal and angular movement. Errors in horizontal velocity were 2.5-fold higher for a 30 than 90 degree camera angle. Differences were small between 60 and 90 degree camera angles, and limited to horizontal velocity. However, errors in both horizontal and angular measures were 2-fold higher for 30 than 90 degree camera angles. Our results agree with previous studies which reported that 90 degree camera angles provided the most accurate horizontal and angular outcomes for Kinovea analysis of body movements in hockey [51], simulated walking [44], and single leg squats [52].

We also examined how the accuracy of Kinovea outcomes differed between 1D versus 2D calibration approaches. We found that the accuracy of outcomes based on 2D calibration decreased with rotation of the calibration grid by 30 degrees or more, and by grid translation of 30 cm or more, indicating the value of accurate positioning of the grid near the plane of the fall. Our 1D calibration results, based on participant height, are valuable for researchers who are unable to duplicate our approach to 2D video calibration, which requires revisiting the scene of the fall and capturing an image of a 1.6 x 1.6 m grid placed in the plane of the fall, with the same (unmoved) camera that recorded the fall. In general, there was little difference between 1D and 2D calibration in the accuracy of vertical outcomes, but errors in horizontal velocity were 1.6-fold higher for 1D than 2D calibration. However, for video collected from cameras oriented at 90 degrees, 1D calibration provided vertical, horizontal and angular velocity measures that were nearly as accurate (to within 4%) as 2D calibration values. Collectively, our results indicate that 1D video calibration based on height was nearly as accurate as 2D (grid) calibration for vertical measures, and for horizontal and angular measures when the camera was oriented at 90 degrees to the plane of the fall.

This study has important limitations. First, all of our data was collected from three healthy young adults, who experienced falls onto a firm gym mat, that were either self-initiated (simulating incorrect weight shifting), or caused by rapid translation of the ground surface. We see no reason why our approaches would not yield similar accuracy in estimating the kinematics of falls in different populations, including older adults. However, movement patterns during falls are diverse, and specific types of falls outside the range we examined (e.g., falls on stairs, falls from an initial sitting position, or falls involving excessive axial rotation during descent) may yield different accuracy from Kinovea. Second, when visible in the video footage, the reflective markers used for 3D motion capture helped in identifying body part locations to track in Kinovea. The lack of distinct landmarks on body parts in real-life falls may affect the accuracy of the measurements. Third, we only examined video footage having a resolution of 640 x 480 pixels, and our results may not reflect the accuracy that may be observed for video of lower or higher resolution. Additionally, we did not correct for camera lens distortion, which may have affected the accuracy of digitized body parts, particularly when they appeared at the edges of the video image, far from the calibration grid. Finally, studies are needed to compare the accuracy of Kinovea to alternative approaches for extracting kinematics from planar video (e.g., DeepLabCut [53]).

In summary, our results demonstrate that Kinovea can be successfully applied to extract whole-body kinematics from video footage of real-life falls, which is increasingly available [37]. However, lower accuracy was observed for angular kinematics of the upper and lower limb in sideways falls, and for horizontal measures from 30 degree cameras. 1D video calibration based on height was nearly as accurate as 2D (grid) calibration for vertical measures, and for horizontal and angular measures from 90 degree camera angles. The acceptability of the observed errors for a given user will depend on the study design and research questions. Our results can guide users in identifying the types of falls, camera angles, calibration techniques, and kinematic outcomes that meet a given desired level of accuracy in Kinovea.

## Supporting information

S1 Table Differences in positions and velocities between Qualisys ground truth signal and Kinovea signals. The table shows differences between Qualisys ground truth signal (Q) and Kinovea signals (K) in (A) vertical, (B) horizontal and (C) angular position and velocity signals. Comparisons show the effects of different (i) cut-off frequencies, (ii) fall directions, (iii-vi) body parts for each fall direction, (vii) camera angles, and (viii-xvii) calibration techniques and orientations. In the camera angles comparisons, Qualisys values (vertical, horizontal, and angular) are different between the 90 degree, and the 60 and 30 degree cameras because markers from the right versus the left side of the body were analyzed, respectively. RMSE and NRMSE are based on comparing signals throughout the fall. Results show mean ± 1 SE values for all falls in a given category. For positions, values less than 0.001 m are rounded up. For velocities, values less than 0.01 m/s are rounded up.
(DOCX)

S2 Table Raw unfiltered position data from Kinovea. The table shows time-varying horizontal (X) and vertical (Z) positions in meters for each body part. Each sheet in the table is a separate fall.
(XLSX)

S3 Table Raw unfiltered position data from Qualisys during backward falls. The table shows time-varying 3D positions (in meters) for all body parts during backward falls. The X axis represents horizontal position in the plane of the fall, Y represents horizontal position perpendicular to the plane of the fall, and Z represent vertical position. Each sheet in the table is a single fall. Frames corresponding to fall initiation and end of the fall are noted.
(XLSX)

S4 Table Raw unfiltered position data from Qualisys during forward falls. The table shows time-varying 3D positions (in meters) for all body parts during forward falls. The X axis represents horizontal position in the plane of the fall, Y represents horizontal position perpendicular to the plane of the fall, and Z represent vertical position. Each sheet in the table is a single fall. Frames corresponding to fall initiation and end of the fall are noted.
(XLSX)

S5 Table Raw unfiltered position data from Qualisys during sideways falls. The table shows time-varying 3D positions (in meters) for all body parts during sideways falls. The X axis represents horizontal position in the plane of the fall, Y represents horizontal position perpendicular to the plane of the fall, and Z represent vertical position. Each sheet in the table is a single fall. Frames corresponding to fall initiation and end of the fall are noted.
(XLSX)

S6 Table Results of the measures of accuracy between Qualisys ground truth signal and Kinovea signals. The table shows measures of accuracy (RMSE, NRMSE and differences in peak velocities) for Kinovea results, used in statistical analyses of the effect of cut-off frequency, fall direction, camera angle, and calibration technique and orientations. Each type of comparison includes separate sheets in the table for vertical, horizontal, and angular measures.
(XLSX)

## References

[1] Canadian Institute for Health Information. Injury and Trauma Emergency Department and Hospitalization Statistics (2016-2017-injury-and-trauma-quick-stats-en-web.xlsx). Canadian Institute for Health Information Access Data and Reports [Internet]; 2016–2017. Available from: https://www.cihi.ca/en/injury-and-trauma-emergency-department-and-hospitalization-statistics-2016-2017.

[2] World Health Organization. Falls [Internet]; 2018. Available from: http://www.who.int/news-room/fact-sheets/detail/falls.

[3] Florence CS, Bergen G, Atherly A, Burns E, Stevens J, Drake C. Medical costs of fatal and nonfatal falls in older adults. J Am Geriatr Soc. 2018;66(4): 693–698. doi: 10.1111/jgs.15304 29512120 PMC6089380

[4] Fu TS, Jing R, Fu WW, Cusimano MD. Epidemiological trends of traumatic brain injury identified in the emergency department in a publicly-insured population, 2002–2010. PloS one. 2016;11(1): e0145469. doi: 10.1371/journal.pone.0145469 26760779 PMC4720113

[5] Parkkari J, Kannus P, Palvanen M, Natri A, Vainio J, Aho H, et al. Majority of hip fractures occur as a result of a fall and impact on the greater trochanter of the femur: a prospective controlled hip fracture study with 206 consecutive patients. Calcif Tissue Int. 1999;65(3): 183–187. doi: 10.1007/s002239900679 10441647

[6] Ioannidis G, Papaioannou A, Hopman WM, Akhtar-Danesh N, Anastassiades T, Pickard L, et al. Relation between fractures and mortality: results from the Canadian Multicentre Osteoporosis Study. CMAJ: Canadian Medical Association journal = journal de l’Association medicale canadienne. 2009;181(5): 265–271. doi: 10.1503/cmaj.081720 19654194 PMC2734204

[7] Sherrington C, Fairhall N, Wallbank G, Tiedemann A, Michaleff ZA, Howard K, et al. Exercise for preventing falls in older people living in the community: an abridged Cochrane systematic review. Br J Sports Med. 2020;54(15): 885–891. doi: 10.1136/bjsports-2019-101512 31792067

[8] Schoberer D, Breimaier HE. Meta-analysis and GRADE profiles of exercise interventions for falls prevention in long-term care facilities. J Adv Nurs. 2020;76(1): 121–134. doi: 10.1111/jan.14238 31642090 PMC6972676

[9] Wang Q, Jiang X, Shen Y, Yao P, Chen J, Zhou Y, et al. Effectiveness of exercise intervention on fall-related fractures in older adults: a systematic review and meta-analysis of randomized controlled trials. BMC Geriatr. 2020;20(1): 322. doi: 10.1186/s12877-020-01721-6 32887571 PMC7650290

[10] Groen BE, Smulders E, De Kam D, Duysens J, Weerdesteyn V. Martial arts fall training to prevent hip fractures in the elderly. Osteoporos Int. 2010;21(2): 215–221. doi: 10.1007/s00198-009-0934-x 19407919

[11] Verrusio W, Gianturco V, Cacciafesta M, Marigliano V, Troisi G, Ripani M. Fall prevention in the young old using an exoskeleton human body posturizer: a randomized controlled trial. Aging Clin Exp Res. 2017;29(2): 207–214. doi: 10.1007/s40520-016-0540-7 26849366

[12] Bateni H, Maki BE. Assistive devices for balance and mobility: benefits, demands, and adverse consequences. Arch Phys Med Rehabil. 2005;86(1): 134–145. doi: 10.1016/j.apmr.2004.04.023 15641004

[13] Monaco V, Tropea P, Aprigliano F, Martelli D, Parri A, Cortese M, et al. An ecologically-controlled exoskeleton can improve balance recovery after slippage. Scientific Reports. 2017;7. doi: 10.1038/srep46721 28492520 PMC5426188

[14] Pynoos J, Steinman BA, Nguyen AQ. Environmental assessment and modification as fall-prevention strategies for older adults. Clin Geriatr Med. 2010;26(4): 633–644. doi: 10.1016/j.cger.2010.07.001 20934614 PMC6036911

[15] Chase CA, Mann K, Wasek S, Arbesman M. Systematic review of the effect of home modification and fall prevention programs on falls and the performance of community-dwelling older adults. Am J Occup Ther. 2012;66(3): 284–291. doi: 10.5014/ajot.2012.005017 22549593

[16] Scanlan A, MacKay M, Olsen L, Clark M, McKim K, Raina P. Sports and Recreation Injury Prevention Strategies: Systematic Review and Best Practices. 2001. Available from: https://citeseerxistpsuedu/viewdoc/download?doi=10111129179&rep=rep1&type=pdf.

[17] Mackey DC, Lachance CC, Wang PT, Feldman F, Laing AC, Leung PM, et al. The Flooring for Injury Prevention (FLIP) Study of compliant flooring for the prevention of fall-related injuries in long-term care: A randomized trial. PLoS medicine. 2019;16(6): e1002843. doi: 10.1371/journal.pmed.1002843 31233541 PMC6590787

[18] Pang I, Okubo Y, Sturnieks D, Lord SR, Brodie MA. Detection of Near Falls Using Wearable Devices: A Systematic Review. J Geriatr Phys Ther. 2019;42(1): 48–56. doi: 10.1519/JPT.0000000000000181 29384813

[19] Rajagopalan R, Litvan I, Jung TP. Fall Prediction and Prevention Systems: Recent Trends, Challenges, and Future Research Directions. Sensors. 2017;17(11): 2509. 10.3390/s17112509 PMC5713074 29104256

[20] Rucco R, Sorriso A, Liparoti M, Ferraioli G, Sorrentino P, Ambrosanio M, et al. Type and Location of Wearable Sensors for Monitoring Falls during Static and Dynamic Tasks in Healthy Elderly: A Review. Sensors. 2018;18(5): 1613. 10.3390/s18051613 PMC5982638 29783647

[21] Chaudhuri S, Thompson H, Demiris G. Fall detection devices and their use with older adults: a systematic review. J Geriatr Phys Ther. 2014;37(4): 178–196. doi: 10.1519/JPT.0b013e3182abe779 24406708 PMC4087103

[22] Geertsema EE, Visser GH, Viergever MA, Kalitzin SN. Automated remote fall detection using impact features from video and audio. J Biomech. 2019;88: 25–32. doi: 10.1016/j.jbiomech.2019.03.007 30922611

[23] Aziz O, Klenk J, Schwickert L, Chiari L, Becker C, Park EJ, et al. Validation of accuracy of SVM-based fall detection system using real-world fall and non-fall datasets. PloS one. 2017;12(7): e0180318. doi: 10.1371/journal.pone.0180318 28678808 PMC5498034

[24] Tamura T, Yoshimura T, Sekine M, Uchida M, Tanaka O. A wearable airbag to prevent fall injuries. IEEE transactions on information technology in biomedicine: a publication of the IEEE Engineering in Medicine and Biology Society. 2009;13(6): 910–914. doi: 10.1109/TITB.2009.2033673 19846379

[25] Post E, Komisar V, Sims-Gould J, Korall A, Feldman F, Robinovitch SN. Development of a stick-on hip protector: A multiple methods study to improve hip protector design for older adults in the acute care environment. J Rehabil Assist Technol Eng. 2019;6: 2055668319877314. doi: 10.1177/2055668319877314 31839983 PMC6896132

[26] Yang Y, Komisar V, Shishov N, Lo B, Korall AM, Feldman F, et al. The Effect of Fall Biomechanics on Risk for Hip Fracture in Older Adults: A Cohort Study of Video-Captured Falls in Long-Term Care. J Bone Miner Res. 2020;35(10): 1914–1922. doi: 10.1002/jbmr.4048 32402136 PMC7689902

[27] Santesso N, Carrasco‐Labra A, Brignardello‐Petersen R. Hip protectors for preventing hip fractures in older people. Cochrane Database of Systematic Reviews. 2014;(3). doi: 10.1002/14651858.CD001255.pub5 24687239 PMC10754476

[28] Korall AMB, Feldman F, Yang Y, Cameron ID, Leung PM, Sims-Gould J, et al. Effectiveness of Hip Protectors to Reduce Risk for Hip Fracture from Falls in Long-Term Care. J Am Med Dir Assoc. 2019;20(11): 1397–1403.e1391. doi: 10.1016/j.jamda.2019.07.010 31477556

[29] Feldman F, Robinovitch SN. Reducing hip fracture risk during sideways falls: evidence in young adults of the protective effects of impact to the hands and stepping. J Biomech. 2007;40(12): 2612–2618. doi: 10.1016/j.jbiomech.2007.01.019 17395188

[30] Wood TA, Moon Y, Sun R, Bishnoi A, Sosnoff JJ. Age-Related Differences in Head Impact during Experimentally Induced Sideways Falls. Biomed Res Int. 2019;2019: 6804614. doi: 10.1155/2019/6804614 31143775 PMC6501264

[31] Choi WJ, Robinovitch SN, Ross SA, Phan J, Cipriani D. Effect of neck flexor muscle activation on impact velocity of the head during backward falls in young adults. Clin Biomech (Bristol, Avon). 2017;49: 28–33. doi: 10.1016/j.clinbiomech.2017.08.007 28843128

[32] Hsiao ET, Robinovitch SN. Common protective movements govern unexpected falls from standing height. J Biomech. 1998;31(1): 1–9. doi: 10.1016/s0021-9290(97)00114-0 9596532

[33] Klenk J, Becker C, Lieken F, Nicolai S, Maetzler W, Alt W, et al. Comparison of acceleration signals of simulated and real-world backward falls. Med Eng Phys. 2011;33(3): 368–373. doi: 10.1016/j.medengphy.2010.11.003 21123104

[34] Kangas M, Vikman I, Nyberg L, Korpelainen R, Lindblom J, Jämsä T. Comparison of real-life accidental falls in older people with experimental falls in middle-aged test subjects. Gait & posture. 2012;35(3): 500–505. doi: 10.1016/j.gaitpost.2011.11.016 22169389

[35] Robinovitch SN, Feldman F, Yang Y, Schonnop R, Leung PM, Sarraf T, et al. Video capture of the circumstances of falls in elderly people residing in long-term care: an observational study. Lancet. 2013;381(9860): 47–54. doi: 10.1016/S0140-6736(12)61263-X 23083889 PMC3540102

[36] Holliday P, Fernie G, Gryfe C, Griggs G. Video recording of spontaneous falls of the elderly. In Slips, stumbles and falls: pedestrian footwear and surfaces. ASTM International. 1990: 7–16.

[37] Robinovitch SN. Falls experienced by older adult residents in long-term care homes. Databrary [Internet]; 2018. Available from 10.17910/b7.739.

[38] Han D, Adolph KE. The impact of errors in infant development: Falling like a baby. Dev Sci. 2020: e13069. 10.1111/desc.13069 PMC8178414 33278863

[39] Robinovitch SN, Hayes WC, McMahon TA. Prediction of femoral impact forces in falls on the hip. J Biomech Eng. 1991;113(4): 366–374. doi: 10.1115/1.2895414 1762432

[40] Kuo C, Shishov N, Elabd K, Komisar V, Chong H, Phu T, et al., editors. Estimating trunk and neck stabilization for avoiding head impact during real-world falls in older adults. 2020 42nd Annual International Conference of the IEEE Engineering in Medicine & Biology Society (EMBC); 2020: IEEE. 4823–4826. 10.1109/EMBC44109.2020.9176257 33019070

[41] Choi WJ, Wakeling JM, Robinovitch SN. Kinematic analysis of video-captured falls experienced by older adults in long-term care. J Biomech. 2015;48(6): 911–920. doi: 10.1016/j.jbiomech.2015.02.025 25769730

[42] Fernández-González P, Koutsou A, Cuesta-Gómez A, Carratalá-Tejada M, Miangolarra-Page JC, Molina-Rueda F. Reliability of Kinovea(®) Software and Agreement with a Three-Dimensional Motion System for Gait Analysis in Healthy Subjects. Sensors 2020;20(11): 3154. doi: 10.3390/s20113154 32498380 PMC7308968

[43] Adnan N, Patar M, Lee H, Yamamoto S-i, Jong-Young L, Mahmud J. Biomechanical analysis using Kinovea for sports application. IOP Conference Series: Materials Science and Engineering. 2018;342: 012097.

[44] Puig-Divi A, Escalona-Marfil C, Padulles-Riu JM, Busquets A, Padulles-Chando X, Marcos-Ruiz D. Validity and reliability of the Kinovea program in obtaining angles and distances using coordinates in 4 perspectives. PloS one. 2019;14(6): e0216448. doi: 10.1371/journal.pone.0216448 31166989 PMC6550386

[45] Aminian K, Najafi B. Capturing human motion using body-fixed sensors: Outdoor measurement and clinical applications. Journal of Visualization and Computer Animation. 2004;15: 79–94.

[46] Pai YC, Patton J. Center of mass velocity-position predictions for balance control. J Biomech. 1997;30(4): 347–354. doi: 10.1016/s0021-9290(96)00165-0 9075002

[47] Yang Y, van Schooten KS, Sims-Gould J, McKay HA, Feldman F, Robinovitch SN. Sex Differences in the Circumstances Leading to Falls: Evidence From Real-Life Falls Captured on Video in Long-Term Care. J Am Med Dir Assoc. 2018;19(2): 130–135.e131. doi: 10.1016/j.jamda.2017.08.011 28967601

[48] Arch Phys Med RehabilJ Biomech Eng Liu J, Lockhart TE. Trunk angular kinematics during slip-induced backward falls and activities of daily living. J Biomech Eng. 2014;136(10): 101005. doi: 10.1115/1.4028033 25033029 PMC4127473

[49] Laing AC, Feldman F, Jalili M, Tsai CM, Robinovitch SN. The effects of pad geometry and material properties on the biomechanical effectiveness of 26 commercially available hip protectors. J Biomech. 2011;44(15): 2627–2635. doi: 10.1016/j.jbiomech.2011.08.016 21899845 PMC3435428

[50] Canadian Standards Association. Hip Protectors (CSA Standard No Z325:20). 2020.

[51] Post A, Koncan D, Kendall M, Cournoyer J, Clark J, Kosziwka G, et al. Analysis of speed accuracy using video analysis software. Sports Eng. 2018;21(3): 235–241.

[52] Schurr SA, Marshall AN, Resch JE, Saliba SA. Two-dimensional video analysis is comparable to 3D motion capture in lower extremity movement assessment. Int J Sports Phys Ther. 2017;12(2): 163–172. 28515970 PMC5380858

[53] Mathis A, Mamidanna P, Cury KM, Abe T, Murthy VN, Mathis MW, et al. DeepLabCut: markerless pose estimation of user-defined body parts with deep learning. Nat Neurosci. 2018;21(9): 1281–1289. doi: 10.1038/s41593-018-0209-y 30127430

