# Supplementary material for: Accuracy of Kinovea software in estimating body segment movements during falls captured on standard video: Effects of fall direction, camera perspective and video calibration technique
Source: PLoS One. 2021 Oct 25;16(10):e0258923. doi: 10.1371/journal.pone.0258923 (PMC8544843; doi:10.1371/journal.pone.0258923)
Supplement: S1 Table — The table shows differences between Qualisys ground truth signal (Q) and Kinovea signals (K) in (A) vertical, (B) horizontal and (C) angular position and velocity signals. Comparisons show the effects of different (i) cut-off frequencies, (ii) fall directions, (iii-vi) body parts for each fall direction, (vii) camera angles, and (viii-xvii) calibration techniques and orientations. In the camera angles comparisons, Qualisys values (vertical, horizontal, and angular) are different between the 90 degree, and the 60 and 30 degree cameras because markers from the right versus the left side of the body were analyzed, respectively. RMSE and NRMSE are based on comparing signals throughout the fall. Results show mean ± 1 SE values for all falls in a given category. For positions, values less than 0.001 m are rounded up. For velocities, values less than 0.01 m/s are rounded up. (DOCX) [file pone.0258923.s001.docx]

| 1. **Vertical position and velocity** | | | | | | | | | | | | |
| --- | --- | --- | --- | --- | --- | --- | --- | --- | --- | --- | --- | --- |
|  | | Position – entire fall | | | Velocity – entire fall | | | Peak velocity | | | | |
|  | | Qualisys 20Hz  Amplitude (m) | RMSE  (m) | NRMSE  (%) | Qualisys 20Hz  Amplitude (m/s) | RMSE  (m/s) | NRMSE  (%) | Qualisys peak  (m/s) | Kinovea peak  (m/s) | Raw difference in peak velocity  (m/s) | Absolute difference in peak velocity (m/s) | Percent difference in peak velocity  (%) |
| (i) Kinovea filter cut-off frequency | 14 Hz | 0.757±0.020 | 0.050±0.002 | 9.2±0.6 | 3.66±0.07 | 0.22±0.01 | 6.5±0.2 | 2.87±0.05 | 2.91±0.06 | -0.04±0.02 | 0.29±0.01 | 1.3±0.9 |
|  | 12 Hz |  | 0.050±0.002 | 9.2±0.6 |  | 0.22±0.01 | 6.5±0.2 |  | 2.90±0.06 | -0.03±0.02 | 0.29±0.02 | -1.1±0.9 |
|  | 10 Hz |  | 0.050±0.002 | 9.2±0.6 |  | 0.22±0.01 | 6.5±0.2 |  | 2.89±0.06 | -0.02±0.02 | 0.28±0.02 | -0.5±0.9 |
|  | 7 Hz |  | 0.050±0.002 | 9.2±0.6 |  | 0.23±0.01 | 6.7±0.2 |  | 2.85±0.06 | 0.03±0.02 | 0.29±0.02 | 1.2±0.9 |
|  | 5 Hz |  | 0.051±0.002 | 9.2±0.6 |  | 0.25±0.01 | 7.2±0.2 |  | 2.76±0.06 | 0.11±0.03 | 0.31±0.02 | 4.3±1.0 |
|  | 3 Hz |  | 0.051±0.002 | 9.4±0.6 |  | 0.32±0.01 | 8.9±0.2 |  | 2.55±0.06 | 0.33±0.03 | 0.41±0.03 | 12.0±1.2 |
| (ii) Fall direction | Backward | 0.801±0.039 | 0.053±0.003 | 8.1±0.7 | 4.31±0.13 | 0.21±0.01 | 5.0±0.2 | 3.24±0.10 | 3.21±0.12 | 0.03±0.04 | 0.31±0.03 | 1.7±1.4 |
|  | Forward | 0.703±0.035 | 0.038±0.003 | 6.9±0.7 | 3.61±0.12 | 0.19±0.01 | 5.4±0.2 | 2.73±0.08 | 2.74±0.10 | -0.01±0.04 | 0.29±0.03 | 0.4±1.5 |
|  | Sideways | 0.765±0.031 | 0.059±0.003 | 12.0±1.3 | 3.13±0.10 | 0.26±0.01 | 8.8±0.4 | 2.67±0.09 | 2.74±0.09 | -0.06±0.03 | 0.26±0.02 | -3.2±1.7 |
| (iii) Body part averaged across all fall directions | Head | 1.109±0.036 | 0.040±0.004 | 3.5±0.3 | 3.67±0.17 | 0.14±0.01 | 4.3±0.4 | 3.04±0.12 | 3.24±0.12 | -0.20±0.03 | 0.20±0.03 | -7.0±1.4 |
|  | Shoulder | 1.006±0.022 | 0.046±0.003 | 4.5±0.3 | 3.56±0.14 | 0.21±0.01 | 6.3±0.5 | 3.08±0.10 | 3.34±0.10 | -0.26±0.04 | 0.28±0.04 | -9.3±1.7 |
|  | Elbow | 0.906±0.017 | 0.068±0.005 | 7.5±0.5 | 3.84±0.13 | 0.26±0..14 | 6.9±0.4 | 3.33±0.10 | 3.43±0.11 | -0.10±0.05 | 0.26±0.03 | -3.4±1.5 |
|  | Wrist | 0.825±0.015 | 0.063±0.005 | 7.5±0.6 | 4.90±0,22 | 0.30±0.01 | 6.3±0.3 | 3.76±0.14 | 3.87±0.16 | -0.11±0.08 | 0.42±0.05 | -3.2±2.3 |
|  | Sternum | 0.954±0.009 | 0.044±0.006 | 4.6±0.6 | 2.99±0.21 | 0.21±0.02 | 7.2±0.5 | 2.65±0.16 | 2.66±0.13 | -0.01±0.08 | 0.17±0.06 | -1.8±3.9 |
|  | ASIS/GT | 0.783±0.021 | 0.046±0.005 | 5.8±0.5 | 3.54±0.14 | 0.18±0.01 | 5.4±0.4 | 2.69±0.10 | 2.65±0.09 | 0.04±0.03 | 0.15±0.11 | 1.1±1.3 |
|  | Knee | 0.402±0.009 | 0.053±0.007 | 13.6±1.8 | 3.04±0.09 | 0.21±0.02 | 7.2±0.5 | 2.19±0.07 | 2.06±0.07 | 0.14±0.04 | 0.20±0.03 | 5.8±1.8 |
|  | Ankle | 0.208±0.024 | 0.038±0.003 | 23.1±2.3 | 3.26±0.29 | 0.25±0.01 | 9.0±0.7 | 2.07±0.17 | 1.71±0.14 | 0.36±0.07 | 0.48±0.05 | 12.9±3.9 |
| (iv) Body part during backward falls | Head | 1.226±0.068 | 0.049±0.008 | 3.8±0.5 | 4.70±0.20 | 0.15±0.01 | 3.2±0.3 | 3.68±0.13 | 3.86±0.14 | -0.18±0.04 | 0.18±0.04 | -4.9±1.1 |
|  | Shoulder | 1.063±0.042 | 0.064±0.006 | 5.9±0.4 | 4.25±0.22 | 0.18±0.01 | 4.4±0.3 | 3.53±0.12 | 3.77±0.15 | -0.24±0.07 | 0.26±0.06 | -6.7±1.8 |
|  | Elbow | 0.959±0.025 | 0.070±0.006 | 7.3±0.5 | 4.49±0.22 | 0.22±0.02 | 5.0±0.3 | 3.67±0.15 | 3.63±0.16 | 0.04±0.09 | 0.23±0.05 | 0.8±2.6 |
|  | Wrist | 0.889±0.023 | 0.086±0.008 | 9.6±0.9 | 5.55±0.35 | 0.33±0.02 | 6.1±0.6 | 4.18±0.26 | 4.19±0.32 | -0.02±0.19 | 0.58±0.09 | -1.0±5.1 |
|  | GT | 0.822±0.017 | 0.039±0.004 | 4.7±0.4 | 4.20±0.22 | 0.16±0.02 | 4.0±0.6 | 3.10±0.15 | 3.12±0.16 | -0.02±0.05 | 0.12±0.03 | -0.7±1.5 |
|  | Knee | 0.362±0.016 | 0.027±0.004 | 7.9±1.6 | 3.27±0.17 | 0.15±0.02 | 4.7±0.5 | 2.19±0.12 | 1.89±0.09 | 0.30±0.08 | 0.32±0.08 | 12.8±3.3 |
|  | Ankle | 0.289±0.062 | 0.037±0.003 | 17.3±2.7 | 3.69±0.50 | 0.26±0.03 | 7.5±0.5 | 2.35±0.32 | 2.04±0.30 | 0.31±0.14 | 0.45±0.09 | 11.7±5.4 |
| (v) Body part during forward falls | Head | 1.096±0.071 | 0.033±0.007 | 2.8±0.5 | 3.38±0.19 | 0.11±0.01 | 3.4±0.4 | 2.83±0.17 | 3.02±0.19 | -0.19±0.04 | 0.19±0.04 | -6.7±1.3 |
|  | Shoulder | 0.940±0.047 | 0.039±0.004 | 4.1±0.3 | 3.33±0.19 | 0.20±0.01 | 6.1±0.5 | 2.89±0.17 | 3.15±0.13 | -0.27±0.06 | 0.29±0.05 | -10.5±2.5 |
|  | Elbow | 0.785±0.015 | 0.062±0.009 | 7.8±1.1 | 3.49±0.18 | 0.22±0.03 | 6.4±0.7 | 2.97±0.16 | 3.21±0.22 | -0.25±0.08 | 0.26±0.07 | -7.5±2.1 |
|  | Wrist | 0.809±0.030 | 0.044±0.007 | 5.3±0.8 | 4.52±0.46 | 0.24±0.03 | 5.2±0.3 | 3.35±0.22 | 3.54±0.29 | -0.19±0.09 | 0.28±0.07 | -4.5±2.7 |
|  | GT | 0.624±0.036 | 0.024±0.004 | 4.2±0.8 | 3.10±0.16 | 0.14±0.01 | 4.5±0.3 | 2.20±0.11 | 2.18±0.08 | 0.01±0.05 | 0.13±0.02 | -0.2±2.4 |
|  | Knee | 0.446±0.011 | 0.027±0.002 | 6.0±0.5 | 2.95±0.16 | 0.17±0.01 | 5.7±0.4 | 2.06±0.10 | 1.90±0.09 | 0.17±0.04 | 0.18±0.04 | 8.0±1.8 |
|  | Ankle | 0.202±0.017 | 0.033±0.005 | 17.8±2.8 | 4.34±0.40 | 0.27±0.02 | 6.4±0.5 | 2.65±0.25 | 2.01±0.20 | 0.64±0.08 | 0.64±0.08 | 24.1±1.9 |
| (vi) Body part during sideways falls | Head | 1.004±0.015 | 0.040±0.007 | 4.0±0.7 | 2.92±0.19 | 0.17±0.02 | 6.2±1.1 | 2.61±0.16 | 2.83±0.17 | -0.22±0.08 | 0.23±0.07 | -9.3±3.7 |
|  | Shoulder | 1.015±0.007 | 0.035±0.003 | 3.4±0.3 | 3.11±0.21 | 0.24±0.02 | 8.4±1.3 | 2.84±0.16 | 3.10±0.15 | -0.27±0.09 | 0.28±0.08 | -10.7±4.0 |
|  | Elbow | 0.975±0.007 | 0.071±0.010 | 7.4±1.0 | 3.55±0.18 | 0.32±0.02 | 9.2±0.6 | 3.35±0.15 | 3.46±0.16 | -0.10±0.10 | 0.29±0.05 | 3.5±2.8 |
|  | Wrist | 0.777±0.016 | 0.059±0.007 | 7.6±1.0 | 4.62±0.27 | 0.34±0.02 | 7.5±0.6 | 3.76±0.17 | 3.88±0.18 | -0.12±0.14 | 0.41±0.07 | -4.0±3.8 |
|  | Sternum | 0.954±0.009 | 0.044±0.006 | 4.6±0.6 | 2.99±0.22 | 0.21±0.02 | 7.2±0.5 | 2.65±0.16 | 2.66±0.13 | -0.01±0.08 | 0.17±0.06 | -1.8±3.9 |
|  | ASIS | 0.862±0.007 | 0.070±0.008 | 8.1±0.9 | 3.22±0.15 | 0.24±0.02 | 7.5±0.5 | 2.64±0.11 | 2.52±0.09 | 0.12±0.06 | 0.21±0.04 | 3.9±2.5 |
|  | Knee | 0.400±0.005 | 0.106±0.007 | 26.8±1.8 | 2.90±0.14 | 0.32±0.02 | 11.1±0.4 | 2.32±0.15 | 2.39±0.14 | -0.06±0.04 | 0.12±0.03 | -3.3±1.9 |
|  | Ankle | 0.132±0.012 | 0.044±0.006 | 34.2±4.2 | 1.75±0.21 | 0.21±0.02 | 13.1±1.3 | 1.22±0.16 | 1.08±0.10 | 0.14±0.11 | 0.34±0.07 | 3.1±9.6 |
| (vii) Camera angle | 90 | 0.757±0.020 | 0.050±0.002 | 9.2±0.6 | 3.66±0.07 | 0.22±0.01 | 6.5±0.2 | 2.87±0.06 | 2.89±0.06 | -0.02±0.02 | 0.28±0.02 | -0.5±0.9 |
|  | 60 | 0.750±0.021 | 0.039±0.002 | 7.1±0.4 | 3.60±0.07 | 0.22±0.01 | 6.3±0.2 | 2.81±0.06 | 2.88±0.07 | -0.07±0.03 | 0.28±0.02 | -2.3±1.0 |
|  | 30 |  | 0.063±0.002 | 12.2±0.8 |  | 0.27±0.01 | 8.2±0.3 |  | 2.97±0.06 | -0.16±0.02 | 0.32±0.02 | -7.3±1.1 |
| (viii) 2D calibration grid translation (averaged across forward and backward translation) | 10cm | 0.757±0.014 | 0.051±0.001 | 9.1±0.4 | 3.66±0.05 | 0.22±0.01 | 6.6±0.1 | 2.87±0.04 | 2.89±0.04 | -0.02±0.02 | 0.29±0.01 | -0.5±0.6 |
|  | 20cm |  | 0.055±0.002 | 9.7±0.4 |  | 0.23±0.01 | 6.8±0.1 |  | 2.89±0.04 | -0.02±0.02 | 0.32±0.01 | -0.5±0.7 |
|  | 30cm |  | 0.061±0.002 | 10.2±0.4 |  | 0.24±0.01 | 7.1±0.1 |  | 2.89±0.04 | -0.02±0.02 | 0.35±0.01 | -0.3±0.7 |
|  | 40cm |  | 0.069±0.002 | 11.3±0.4 |  | 0.26±0.01 | 7.5±0.1 |  | 2.91±0.05 | -0.03±0.02 | 0.41±0.01 | -1.0±0.8 |
|  | 50cm |  | 0.079±0.084 | 12.5±0.4 |  | 0.28±0.01 | 8.0±0.1 |  | 2.91±0.01 | -0.03±0.03 | 0.48±0.01 | -1.2±0.9 |
| (ix) 2D calibration grid rotation (averaged across clockwise and counter clockwise directions) | 15deg |  | 0.090±0.002 | 16.2±0.8 |  | 0.27±0.01 | 8.2±0.2 |  | 2.92±0.04 | -0.04±0.02 | 0.40±0.01 | -1.8±0.9 |
|  | 30deg |  | 0.163±0.005 | 29.3±1.3 |  | 0.39±0.01 | 11.9±0.3 |  | 3.05±0.05 | -0.17±0.04 | 0.66±0.03 | -7.1±1.4 |
|  | 45deg |  | 0.276±0.010 | 48.6±2.3 |  | 0.61±0.02 | 18.5±0.6 |  | 3.36±0.09 | -0.49±0.08 | 1.17±0.06 | -19.9±2.8 |
| (x) 2D calibration grid in the plane of the fall | | 0.757±0.020 | 0.050±0.002 | 9.2±0.6 | 3.66±0.07 | 0.22±0.01 | 6.5±0.2 | 2.87±0.05 | 2.89±0.06 | -0.02±0.02 | 0.28±0.02 | -0.5±0.9 |
| (xi) Forward translation of 2D calibration grid | 10cm |  | 0.043±0.002 | 8.2±0.6 |  | 0.21±0.01 | 6.3±0.2 |  | 2.81±0.06 | 0.06±0.02 | 0.27±0.02 | 2.2±0.9 |
|  | 20cm |  | 0.042±0.002 | 7.7±0.5 |  | 0.21±0.01 | 6.2±0.2 |  | 2.72±0.06 | 0.15±0.02 | 0.28±0.02 | 5.6±0.8 |
|  | 30cm |  | 0.043±0.002 | 7.6±0.5 |  | 0.22±0.01 | 6.4±0.2 |  | 2.64±0.06 | 0.24±0.02 | 0.31±0.02 | 8.4±0.8 |
|  | 40cm |  | 0.049±0.002 | 8.4±0.5 |  | 0.23±0.01 | 6.7±0.2 |  | 2.58±0.06 | 0.29±0.02 | 0.36±0.02 | 10.3±0.8 |
|  | 50cm |  | 0.058±0.003 | 9.3±0.5 |  | 0.24±0.01 | 7.1±0.2 |  | 2.51±0.05 | 0.37±0.02 | 0.42±0.02 | 12.9±0.8 |
| (xii) Backward translation of 2D calibration grid | 10cm |  | 0.058±0.002 | 10.0±0.5 |  | 0.23±0.01 | 6.8±0.2 |  | 2.97±0.06 | -0.10±0.02 | 0.30±0.02 | -3.1±0.9 |
|  | 20cm |  | 0.069±0.002 | 11.7±0.6 |  | 0.25±0.01 | 7.3±0.2 |  | 3.06±0.06 | -0.19±0.03 | 0.36±0.02 | -6.6±1.0 |
|  | 30cm |  | 0.079±0.003 | 12.8±0.6 |  | 0.27±0.01 | 7.7±0.2 |  | 3.14±0.07 | -0.27±0.02 | 0.40±0.02 | -9.0±1.0 |
|  | 40cm |  | 0.090±0.003 | 14.3±0.7 |  | 0.29±0.01 | 8.4±0.2 |  | 3.23±0.07 | -0.36±0.03 | 0.47±0.02 | -12.4±1.0 |
|  | 50cm |  | 0.100±0.003 | 15.7±0.7 |  | 0.31±0.01 | 9.0±0.2 |  | 3.32±0.07 | -0.45±0.03 | 0.53±0.02 | -15.3±1.0 |
| (xiii) Clockwise rotation of 2D calibration grid | 15deg |  | 0.091±0.003 | 16.4±1.1 |  | 0.27±0.01 | 8.1±0.2 |  | 2.85±0.06 | 0.02±0.03 | 0.39±0.02 | 0.7±1.1 |
|  | 30deg |  | 0.157±0.006 | 28.4±1.9 |  | 0.38±0.01 | 11.4±0.4 |  | 2.90±0.07 | -0.03±0.05 | 0.62±0.03 | -2.0±1.8 |
|  | 45deg |  | 0.258±0.012 | 46.9±3.3 |  | 0.57±0.02 | 17.2±0.7 |  | 3.03±0.10 | -0.16±0.09 | 1.03±0.06 | -8.5±3.3 |
| (xiv) Counter clockwise rotation of 2D calibration grid | 15deg |  | 0.089±0.003 | 16.0±1.0 |  | 0.28±0.01 | 8.3±0.3 |  | 2.98±0.07 | -0.11±0.03 | 0.40±0.02 | -4.3±1.3 |
|  | 30deg |  | 0.169±0.007 | 30.1±1.9 |  | 0.40±0.01 | 12.3±0.5 |  | 3.19±0.08 | -0.32±0.06 | 0.70±0.04 | -12.2±2.1 |
|  | 45deg |  | 0.295±0.017 | 50.3±3.2 |  | 0.64±0.03 | 19.8±1.0 |  | 3.70±0.14 | -0.83±0.12 | 1.31±0.10 | -31.3±4.4 |
| (xv) 1D calibration based on height | |  | 0.042±0.002 | 7.3±0.4 |  | 0.23±0.01 | 6.8±0.2 |  | 2.60±0.06 | 0.28±0.02 | 0.37±0.02 | 9.9±0.9 |
| (xvi) 1D calibration based on height + 10cm | |  | 0.050±0.002 | 8.3±0.4 |  | 0.23±0.01 | 6.8±0.2 |  | 2.75±0.06 | 0.12±0.03 | 0.34±0.02 | 4.6±1.0 |
| (xvii) 1D calibration based on height -10cm | |  | 0.046±0.002 | 7.8±0.4 |  | 0.25±0.01 | 7.1±0.2 |  | 2.44±0.05 | 0.43±0.02 | 0.47±0.02 | 15.3±0.9 |

| 1. **Horizontal position and velocity** | | | | | | | | | | | | | | | | | | | | | | | |
| --- | --- | --- | --- | --- | --- | --- | --- | --- | --- | --- | --- | --- | --- | --- | --- | --- | --- | --- | --- | --- | --- | --- | --- |
|  | | Position – entire fall | | | | | | Velocity – entire fall | | | | | | Peak velocity | | | | | | | | | |
|  | | Qualisys 20Hz  Amplitude (m) | | RMSE  (m) | | NRMSE  (%) | | Qualisys 20Hz  Amplitude (m/s) | | RMSE  (m/s) | | NRMSE  (%) | | Qualisys peak  (m/s) | | Kinovea peak  (m/s) | | Raw difference in peak velocity  (m/s) | | Absolute difference in peak velocity (m/s) | | Percent difference in peak velocity  (%) | |
| (i) Kinovea filter cut-off frequency | 14 Hz | 0.709±0.027 | 0.035±0.002 | | 6.0±0.3 | | 2.45±0.06 | | 0.16±0.01 | | 7.3±0.2 | | 1.95±0.05 | | 1.93±0.05 | | 0.03±0.02 | | 0.21±0.01 | | 0.9±1.0 | |  |
|  | 12 Hz |  | 0.035±0.002 | | 6.0±0.3 | |  |  | 0.16±0.01 | | 7.2±0.2 | |  |  | 1.92±0.05 | | 0.03±0.02 | | 0.21±0.01 | | 1.2±1.0 | |  |
|  | 10 Hz |  | 0.035±0.002 | | 6.0±0.3 | |  |  | 0.16±0.01 | | 7.2±0.2 | |  |  | 1.91±0.05 | | 0.05±0.02 | | 0.21±0.01 | | 2.1±1.0 | |  |
|  | 7 Hz |  | 0.035±0.002 | | 6.0±0.3 | |  |  | 0.16±0.01 | | 7.2±0.2 | |  |  | 1.85±0.05 | | 0.10±0.02 | | 0.21±0.01 | | 5.0±0.9 | |  |
|  | 5 Hz |  | 0.035±0.002 | | 6.0±0.3 | |  |  | 0.17±0.01 | | 7.7±0.2 | |  |  | 1.77±0.05 | | 0.18±0.02 | | 0.25±0.02 | | 9.6±0.9 | |  |
|  | 3 Hz |  | 0.036±0.001 | | 6.4±0.3 | |  |  | 0.22±0.01 | | 9.8±0.2 | |  |  | 1.58±0.05 | | 0.37±0.02 | | 0.40±0.02 | | 19.5±1.0 | |  |
| (ii) Fall direction | Backward | 0.678±0.041 | 0.043±0.003 | | 7.1±0.4 | | 2.67±0.12 | | 0.15±0.01 | | 6.4±0.3 | | 2.07±0.10 | | 2.09±0.10 | | -0.03±0.03 | | 0.19±0.02 | | -2.4±1.3 | |  |
|  | Forward | 0.799±0.056 | 0.032±0.003 | | 4.3±0.2 | | 2.64±0.09 | | 0.14±0.01 | | 5.6±0.2 | | 2.08±0.09 | | 2.09±0.10 | | -0.01±0.03 | | 0.18±0.02 | | 0.8±1.4 | |  |
|  | Sideways | 0.661±0.042 | 0.031±0.002 | | 6.5±0.6 | | 2.08±0.08 | | 0.17±0.01 | | 9.2±0.5 | | 1.74±0.07 | | 1.59±0.06 | | 0.16±0.04 | | 0.25±0.03 | | 7.1±1.9 | |  |
| (iii) Body part averaged across all fall directions | Head | 0.976±0.086 | 0.034±0.004 | | 4.6±0.7 | | 2.56±0.13 | | 0.12±0.01 | | 4.9±0.3 | | 2.26±0.14 | | 2.23±0.14 | | 0.03±0.02 | | 0.10±0.02 | | 1.2±1.2 | |  |
|  | Shoulder | 0.865±0.081 | 0.042±0.004 | | 5.7±0.5 | | 2.21±0.13 | | 0.16±0.01 | | 8.0±0.5 | | 1.96±0.14 | | 1.90±0.13 | | 0.06±0.04 | | 0.16±0.03 | | 2.2±1.8 | |  |
|  | Elbow | 0.755±0.075 | 0.053±0.005 | | 8.1±0.6 | | 2.69±0.13 | | 0.21±0.01 | | 8.3±0.5 | | 2.07±0.12 | | 1.93±0.14 | | 0.14±0.07 | | 0.34±0.05 | | 6.6±3.3 | |  |
|  | Wrist | 0.767±0.070 | 0.051±0.005 | | 7.4±0.5 | | 2.98±0.15 | | 0.20±0.01 | | 7.0±0.4 | | 2.31±0.10 | | 2.35±0.11 | | -0.04±0.08 | | 0.31±-0.06 | | -3.0±2.9 | |  |
|  | Sternum | 0.852±0.126 | 0.0137±0.001 | | 2.3±0.4 | | 1.89±0.18 | | 0.16±0.02 | | 10.1±1.9 | | 1.80±0.16 | | 1.65±0.15 | | 0.16±0.05 | | 0.18±0.04 | | 8.6±2.8 | |  |
|  | ASIS/GT | 0.505±0.064 | 0.030±0.003 | | 9.0±1.4 | | 1.67±0.09 | | 0.13±0.01 | | 8.5±0.8 | | 1.24±0.08 | | 1.17±0.08 | | 0.06±0.03 | | 0.14±0.02 | | 4.4±2.4 | |  |
|  | Knee | 0.487±0.047 | 0.023±0.002 | | 5.2±0.3 | | 2.44±0.10 | | 0.13±0.01 | | 5.4±0.3 | | 1.76±0.09 | | 1.70±0.11 | | 0.07±0.04 | | 0.19±0.03 | | 4.9±2.5 | |  |
|  | Ankle | 0.543±0.037 | 0.019±0.002 | | 3.5±0.3 | | 2.69±0.23 | | 0.16±0.01 | | 7.2±0.7 | | 2.06±0.17 | | 2.09±0.17 | | -0.03±0.05 | | 0.22±0.03 | | -4.0±3.1 | |  |
| (iv) Body part during backward falls | Head | 0.847±0.148 | 0.053±0.005 | | 8.7±1.5 | | 2.46±0.29 | | 0.12±0.01 | | 5.4±0.4 | | 2.14±0.31 | | 2.2±0.31 | | -0.02±0.04 | | 0.11±0.03 | | -2.0±1.8 | |  |
|  | Shoulder | 0.773±0.129 | 0.055±0.006 | | 8.5±0.9 | | 2.37±0.30 | | 0.15±0.01 | | 7.0±0.7 | | 2.06±0.30 | | 1.95±0.25 | | 0.11±0.08 | | 0.20±0.05 | | 0.6±3.8 | |  |
|  | Elbow | 0.696±0.110 | 0.053±0.007 | | 8.0±0.5 | | 2.39±0.19 | | 0.17±0.01 | | 7.9±1.1 | | 1.87±0.15 | | 1.76±0.14 | | 0.11±0.09 | | 0.21±0.08 | | 4.1±4.3 | |  |
|  | Wrist | 0.710±0.099 | 0.057±0.008 | | 8.1±0.8 | | 2.88±0.27 | | 0.23±0.02 | | 8.3±0.7 | | 2.16±0.12 | | 2.40±0.11 | | -0.24±0.08 | | 0.32±0.05 | | -12.3±3.9 | |  |
|  | GT | 0.475±0.100 | 0.028±0.005 | | 7.2±0.8 | | 1.66±0.12 | | 0.12±0.01 | | 7.1±0.5 | | 1.17±0.12 | | 1.18±0.10 | | -0.01±0.04 | | 0.11±0.02 | | -3.0±3.1 | |  |
|  | Knee | 0.531±0.074 | 0.027±0.003 | | 5.5±0.6 | | 2.71±0.12 | | 0.12±0.01 | | 4.3±0.3 | | 1.96±0.09 | | 1.98±0.11 | | -0.02±0.04 | | 0.10±0.02 | | -0.8±1.8 | |  |
|  | Ankle | 0.709±0.067 | 0.025±0.004 | | 3.4±0.3 | | 4.20±0.34 | | 0.18±0.02 | | 4.4±0.4 | | 3.14±0.25 | | 3.24±0.26 | | -0.10±0.09 | | 0.27±0.06 | | -3.6±2.9 | |  |
| (v) Body part during forward falls | Head | 1.144±0.176 | 0.021±0.003 | | 2.2±0.3 | | 2.65±0.27 | | 0.10±0.01 | | 4.1±0.3 | | 2.40±0.26 | | 2.36±0.26 | | 0.03±0.04 | | 0.10±0.03 | | 2.1±2.2 | |  |
|  | Shoulder | 0.978±0.166 | 0.031±0.006 | | 3.1±0.3 | | 2.11±0.17 | | 0.14±0.01 | | 7.1±0.5 | | 1.85±0.20 | | 1.92±0.23 | | -0.07±0.06 | | 0.12±0.05 | | -3.3±2.3 | |  |
|  | Elbow | 0.891±0.151 | 0.054±0.008 | | 6.4±0.4 | | 3.22±0.23 | | 0.20±0.01 | | 6.4±0.4 | | 2.24±0.25 | | 2.46±0.27 | | -0.22±0.05 | | 0.24±0.03 | | -10.3±2.6 | |  |
|  | Wrist | 0.894±0.144 | 0.055±0.008 | | 6.7±0.5 | | 3.24±0.24 | | 0.17±0.01 | | 5.4±0.4 | | 2.62±0.21 | | 2.77±0.23 | | -0.15±0.06 | | 0.23±0.03 | | -5.6±2.2 | |  |
|  | GT | 0.569±0.138 | 0.021±0.006 | | 3.8±0.5 | | 2.18±0.17 | | 0.11±0.01 | | 5.2±0.5 | | 1.63±0.14 | | 1.57±0.17 | | 0.07±0.06 | | 0.15±0.03 | | 5.9±4.0 | |  |
|  | Knee | 0.525±0.092 | 0.020±0.003 | | 4.4±0.5 | | 2.63±0.19 | | 0.14±0.01 | | 5.4±0.5 | | 1.94±0.22 | | 1.76±0.27 | | 0.18±0.08 | | 0.25±0.06 | | 13.0±4.5 | |  |
|  | Ankle | 0.531±0.052 | 0.018±0.004 | | 3.3±0.6 | | 2.35±0.16 | | 0.13±0.01 | | 5.6±0.5 | | 1.78±0.15 | | 1.67±0.13 | | 0.12±0.06 | | 0.19±0.04 | | 4.7±4.1 | |  |
| (vi) Body part during sideways falls | Head | 0.936±0.115 | 0.029±0.007 | | 2.9±0.6 | | 2.57±0.13 | | 0.13±0.02 | | 5.2±0.6 | | 2.24±0.13 | | 2.16±0.13 | | 0.07±0.04 | | 0.11±0.04 | | 3.5±2.2 | |  |
|  | Shoulder | 0.843±0.128 | 0.040±0.006 | | 5.6±0.8 | | 2.16±0.21 | | 0.20±0.02 | | 9.8±0.9 | | 1.98±0.21 | | 1.84±0.23 | | 0.15±0.03 | | 0.16±0.02 | | 9.4±2.1 | |  |
|  | Elbow | 0.679±0.127 | 0.051±0.009 | | 9.8±1.5 | | 2.46±0.20 | | 0.25±0.02 | | 10.6±0.8 | | 2.10±0.23 | | 1.56±0.21 | | 0.54±0.12 | | 0.57±0.11 | | 25.9±4.6 | |  |
|  | Wrist | 0.696±0.118 | 0.042±0.009 | | 7.3±1.1 | | 2.83±0.29 | | 0.20±0.02 | | 7.2±0.7 | | 2.16±0.16 | | 1.88±0.10 | | 0.28±0.18 | | 0.39±0.16 | | 8.9±6.2 | |  |
|  | Sternum | 0.852±0.126 | 0.014±0.001 | | 2.3±0.4 | | 1.89±0.18 | | 0.16±0.02 | | 10.1±1.9 | | 1.80±0.16 | | 1.65±0.15 | | 0.16±0.05 | | 0.18±0.04 | | 8.6±2.8 | |  |
|  | ASIS | 0.487±0.110 | 0.038±0.005 | | 14.6±3.1 | | 1.29±0.07 | | 0.15±0.01 | | 12.4±1.4 | | 1.01±0.12 | | 0.87±0.09 | | 0.14±0.05 | | 0.17±0.03 | | 10.7±4.3 | |  |
|  | Knee | 0.405±0.078 | 0.021±0.004 | | 5.5±0.5 | | 1.99±0.11 | | 0.13±0.01 | | 6.7±0.6 | | 1.40±0.05 | | 1.36±0.09 | | 0.04±0.08 | | 0.23±0.03 | | 2.7±5.4 | |  |
|  | Ankle | 0.390±0.036 | 0.014±0.001 | | 3.7±0.5 | | 1.51±0.18 | | 0.16±0.02 | | 11.6±1.4 | | 1.25±0.13 | | 1.36±0.12 | | -0.11±0.07 | | 0.21±0.05 | | -13.0±7.3 | |  |
| (vii) Camera angle | 90 | 0.709±0.027 | 0.035±0.002 | | 6.0±0.3 | | 2.45±0.06 | | 0.16±0.01 | | 7.2±0.2 | | 1.95±0.05 | | 1.91±0.05 | | 0.05±0.02 | | 0.21±0.01 | | 2.1±1.0 | |  |
|  | 60 | 0.718±0.028 | 0.037±0.002 | | 9.0±0.7 | | 2.40±0.06 | | 0.21±0.01 | | 9.5±0.3 | | 1.97±0.05 | | 1.89±0.05 | | 0.07±0.02 | | 0.27±0.02 | | 1.9±1.2 | |  |
|  | 30 |  | 0.098±0.004 | | 25.2±2.1 | |  |  | 0.40±0.01 | | 19.3±0.8 | |  |  | 2.08±0.05 | | -0.12±0.04 | | 0.50±0.03 | | -13.2±2.5 | |  |
| (viii) 2D calibration grid translation (averaged across forward and backward translation) | 10cm | 0.709±0.019 | 0.037±0.001 | | 6.1±0.2 | | 2.45±0.04 | | 0.16±0.01 | | 7.3±0.2 | | 1.95±0.03 | | 1.91±0.04 | | 0.04±0.01 | | 0.22±0.01 | | 1.7±0.7 | |  |
|  | 20cm |  | 0.040±0.002 | | 6.5±0.2 | |  |  | 0.17±0.01 | | 7.5±0.2 | |  |  | 1.91±0.04 | | 0.04±0.01 | | 0.23±0.01 | | 1.7±0.7 | |  |
|  | 30cm |  | 0.046±0.002 | | 7.4±0.2 | |  |  | 0.17±0.01 | | 7.8±0.2 | |  |  | 1.91±0.04 | | 0.04±0.02 | | 0.26±0.01 | | 1.7±0.8 | |  |
|  | 40cm |  | 0.055±0.002 | | 8.6±0.2 | |  |  | 0.18±0.01 | | 8.2±0.2 | |  |  | 1.92±0.04 | | 0.03±0.02 | | 0.29±0.01 | | 1.2±0.8 | |  |
|  | 50cm |  | 0.065±0.002 | | 9.8±0.3 | |  |  | 0.19±0.01 | | 8.7±0.2 | |  |  | 1.92±0.04 | | 0.03±0.02 | | 0.33±0.01 | | 1.1±0.9 | |  |
| (ix) 2D calibration grid rotation (averaged across clockwise and counter clockwise directions) | 15deg |  | 0.059±0.002 | | 9.0±0.2 | |  |  | 0.19±0.01 | | 8.5±0.2 | |  |  | 1.99±0.04 | | -0.04±0.02 | | 0.31±0.01 | | -2.9±0.9 | |  |
|  | 30deg |  | | 0.127±0.006 | | 17.9±0.5 | |  | | 0.31±0.01 | | 13.1±0.3 | |  | | 2.34±0.05 | | -0.39±0.04 | | 0.63±0.03 | | -20.1±1.5 | |
|  | 45deg |  |  | 0.277±0.016 | | 36.1±1.2 | |  |  | 0.59±0.03 | | 24.3±0.9 | |  |  | 3.18±0.11 | | -1.23±0.09 | | 1.44±0.09 | | -59.1±3.7 | |
| (x) 2D calibration grid in the plane of the fall | | 0.709±0.027 | | 0.035±0.002 | | 6.0±0.3 | | 2.45±0.06 | | 0.16±0.01 | | 7.2±0.2 | | 1.95±0.05 | | 1.91±0.05 | | 0.05±0.02 | | 0.21±0.01 | | 2.1±1.0 | |
| (xi) Forward translation of 2D calibration grid | 10cm |  |  | 0.031±0.001 | | 5.4±0.3 | |  |  | 0.15±0.01 | | 7.0±0.2 | |  |  | 1.86±0.05 | | 0.10±0.02 | | 0.22±0.01 | | 4.5±0.9 | |
|  | 20cm |  |  | 0.030±0.001 | | 5.4±0.3 | |  |  | 0.15±0.01 | | 7.0±0.2 | |  |  | 1.80±0.05 | | 0.15±0.02 | | 0.23±0.01 | | 7.6±0.9 | |
|  | 30cm |  |  | 0.031±0.002 | | 5.5±0.3 | |  |  | 0.16±0.01 | | 7.0±0.2 | |  |  | 1.74±0.05 | | 0.21±0.02 | | 0.26±0.02 | | 10.4±0.9 | |
|  | 40cm |  |  | 0.039±0.002 | | 6.6±0.3 | |  |  | 0.16±0.01 | | 7.3±0.2 | |  |  | 1.71±0.05 | | 0.25±0.02 | | 0.29±0.02 | | 12.4±0.9 | |
|  | 50cm |  |  | 0.048±0.002 | | 7.7±0.3 | |  |  | 0.17±0.01 | | 7.7±0.2 | |  |  | 1.66±0.05 | | 0.29±0.02 | | 0.33±0.02 | | 14.9±0.9 | |
| (xii) Backward translation of 2D calibration grid | 10cm |  |  | 0.042±0.002 | | 6.8±0.3 | |  |  | 0.17±0.30 | | 7.5±0.2 | |  |  | 1.96±0.05 | | -0.01±0.02 | | 0.22±0.01 | | -1.1±1.0 | |
|  | 20cm |  |  | 0.051±0.002 | | 7.7±0.3 | |  |  | 0.18±0.01 | | 8.0±0.2 | |  |  | 2.02±0.05 | | -0.07±0.02 | | 0.24±0.01 | | -4.1±1.0 | |
|  | 30cm |  |  | 0.061±0.003 | | 9.3±0.3 | |  |  | 0.19±0.01 | | 8.5±0.2 | |  |  | 2.08±0.06 | | -0.13±0.00 | | 0.26±0.02 | | -7.1±1.0 | |
|  | 40cm |  |  | 0.072±0.003 | | 10.7±0.4 | |  |  | 0.20±0.01 | | 9.0±0.2 | |  |  | 2.14±0.06 | | -0.18±0.02 | | 0.29±0.02 | | -10.1±1.1 | |
|  | 50cm |  |  | 0.081±0.004 | | 12.0±0.4 | |  |  | 0.22±0.01 | | 9.6±0.2 | |  |  | 2.19±0.06 | | -0.23±0.02 | | 0.33±0.02 | | -12.7±1.1 | |
| (xiii) Clockwise rotation of 2D calibration grid | 15deg |  |  | 0.066±0.004 | | 9.1±0.3 | |  |  | 0.22±0.01 | | 8.7±0.2 | |  |  | 1.93±0.06 | | 0.02±0.03 | | 0.37±0.02 | | 0.7±1.3 | |
|  | 30deg |  |  | 0.122±0.008 | | 15.6±0.6 | |  |  | 0.30±0.01 | | 12.2±0.4 | |  |  | 2.19±0.08 | | -0.24±0.05 | | 0.63±0.04 | | -10.8±2.1 | |
|  | 45deg |  |  | 0.238±0.019 | | 28.9±1.3 | |  |  | 0.52±0.03 | | 20.3±1.2 | |  |  | 2.79±0.14 | | -0.84±0.11 | | 1.21±0.10 | | -36.6±4.1 | |
| (xiv) Counter clockwise rotation of 2D calibration grid | 15deg |  |  | 0.051±0.002 | | 8.8±0.4 | |  |  | 0.18±0.01 | | 8.3±0.2 | |  |  | 2.04±0.05 | | -0.09±0.02 | | 0.26±0.02 | | -6.5±1.1 | |
|  | 30deg |  |  | 0.132±0.008 | | 20.1±0.7 | |  |  | 0.32±0.01 | | 14.0±0.5 | |  |  | 2.48±0.07 | | -0.53±0.04 | | 0.63±0.04 | | -29.4±2.0 | |
|  | 45deg |  |  | 0.314±0.024 | | 43.3±2.0 | |  |  | 0.66±0.04 | | 28.3±1.5 | |  |  | 3.57±0.17 | | -1.62±0.11 | | 1.68±0.15 | | -81.7±5.9 | |
| (xv) 1D calibration based on height | |  |  | 0.108±0.004 | | 16.9±0.5 | |  |  | 0.26±0.01 | | 11.2±0.3 | |  |  | 1.35±0.03 | | 0.60±0.02 | | 0.61±0.02 | | 29.6±0.8 | |
| (xvi) 1D calibration based on height + 10cm | |  |  | 0.091±0.004 | | 14.7±0.6 | |  |  | 0.23±0.01 | | 10.3±0.3 | |  |  | 1.43±0.04 | | 0.52±0.02 | | 0.53±0.02 | | 25.4±0.9 | |
| (xvii) 1D calibration based on height -10cm | |  |  | 0.125±0.005 | | 19.2±0.5 | |  |  | 0.28±0.01 | | 12.2±0.3 | |  |  | 1.27±0.03 | | 0.68±0.02 | | 0.68±0.02 | | 33.7±0.8 | |

| **(C) Angular position and velocity** | | | | | | | | | | | |
| --- | --- | --- | --- | --- | --- | --- | --- | --- | --- | --- | --- |
|  | | Position – entire fall | | | Velocity – entire fall | | | Peak velocity | | | |
|  | | Qualisys 20Hz  Amplitude (deg) | RMSE  (deg) | NRMSE  (%) | Qualisys 20Hz  Amplitude (deg/s) | RMSE  (deg/s) | NRMSE  (%) | Qualisys peak  (deg/s) | Kinovea peak  (deg/s) | Raw difference in peak velocity  (deg/s) | Absolute difference in peak velocity  (deg/s) |
| (i) Kinovea filter cut-off frequency | 14 Hz | 79.5±2.5 | 10.1±0.9 | 13.0±0.9 | 631±23 | 63±4 | 10.0±0.4 | 462±19 | 354±11 | 108±18 | 145±16 |
|  | 12 Hz |  | 10.1±0.9 | 13.0±0.9 |  | 63±4 | 10.0±0.4 |  | 352±11 | 110±18 | 146±16 |
|  | 10 Hz |  | 10.1±0.9 | 13.0±0.9 |  | 63±4 | 10.0±0.4 |  | 347±11 | 115±18 | 148±16 |
|  | 7 Hz |  | 10.1±0.9 | 13.0±0.9 |  | 65±4 | 10.2±0.4 |  | 332±11 | 130±18 | 155±17 |
|  | 5 Hz |  | 10.1±0.9 | 13.1±0.9 |  | 69±4 | 10.8±0.3 |  | 307±10 | 155±18 | 170±17 |
|  | 3 Hz |  | 10.4±0.9 | 13.5±0.9 |  | 79±4 | 12.6±0.3 |  | 258±9 | 204±18 | 209±18 |
| (ii) Fall direction | Backward | 82.6±4.7 | 3.9±1.2 | 6.2±0.7 | 623±33 | 40±6 | 6.7±0.3 | 443±24 | 395±24 | 48±10 | 75±7 |
|  | Forward | 73.7±3.8 | 4.9±1.2 | 7.2±0.6 | 604±27 | 45±6 | 7.6±0.3 | 415±22 | 332±17 | 83±14 | 97±12 |
|  | Sideways | 82.2±4.2 | 21.5±1.2 | 25.6±1.9 | 667±55 | 104±6 | 15.6±0.7 | 528±45 | 314±15 | 214±48 | 272±44 |
| (iii) Body part averaged across all fall directions | Head | 50.9±4.6 | 8.8±1.0 | 23.4±3.3 | 539±50 | 47±3 | 10.9±1.2 | 415±44 | 319±28 | 96±25 | 135±19 |
|  | Forearm | 84.4±7.0 | 14.5±2.9 | 13.1±1.8 | 845±64 | 89±12 | 10.0±0.7 | 602±59 | 370±29 | 232±58 | 243±56 |
|  | Upper arm | 69.3±5.7 | 7.0±1.0 | 10.2±1.0 | 611±29 | 63±6 | 10.4±0.8 | 424±26 | 349±28 | 75±20 | 110±15 |
|  | Torso | 70.1±3.6 | 3.2±0.3 | 4.8±0.4 | 372±23 | 29±2 | 8.4±0.6 | 297±19 | 275±18 | 22±8 | 42±5 |
|  | Thigh | 105.9±6.3 | 17.1±3.6 | 15.6±3.1 | 801±76 | 92±17 | 9.4±0.9 | 584±63 | 305±18 | 279±70 | 295±68 |
|  | Shank | 96.4±3.1 | 9.9±1.3 | 11.0±1.6 | 620±43 | 57±4 | 10.7±1.1 | 449±25 | 464±31 | -16±14 | 63±10 |
| (iv) Body part during backward falls | Head | 52.4±9.4 | 5.0±0.5 | 14.0±3.5 | 578±88 | 36±4 | 7.0±0.7 | 416±74 | 315±55 | 101±22 | 102±21 |
|  | Forearm | 70.5±7.3 | 3.4±0.4 | 5.2±0.6 | 819±82 | 48±4 | 6.0±0.3 | 5503±57 | 468±56 | 82±23 | 83±22 |
|  | Upper arm | 65.9±7.1 | 3.7±0.5 | 6.1±0.8 | 598±47 | 44±3 | 7.6±0.6 | 435±49 | 327±46 | 108±11 | 108±11 |
|  | Torso | 66.6±9.0 | 2.5±0.4 | 4.1±0.4 | 389±50 | 25±2 | 6.7±0.4 | 309±51 | 269±40 | 40±15 | 48±12 |
|  | Thigh | 133.5±8.9 | 3.2±0.3 | 2.6±0.4 | 551±54 | 35±5 | 6.4±0.6 | 369±25 | 349±20 | 21±9 | 31.89±.8 |
|  | Shank | 106.8±8.0 | 5.6±0.7 | 5.5±0.7 | 802±91 | 52±9 | 6.7±1.0 | 575±53 | 641±59 | -66±25 | 75±23 |
| (v) Body part during forward falls | Head | 65.4±8.3 | 5.6±0.8 | 9.5±1.3 | 724±82 | 51±5 | 7.4±0.4 | 591±81 | 354±54 | 237±40 | 237±40 |
|  | Forearm | 50.6±5.3 | 4.0±0.8 | 7.5±1.1 | 636±71 | 52±5 | 8.5±0.4 | 379±42 | 348±50 | 31±19 | 60±12 |
|  | Upper arm | 77.9±15.3 | 10.2±2.4 | 14.5±1.9 | 636±74 | 63±10 | 10.2±1.0 | 445±61 | 308±42 | 137±32 | 140±30 |
|  | Torso | 81.9±4.3 | 2.5±0.4 | 3.0±0.3 | 402±45 | 26±2 | 6.9±0.6 | 299±28 | 265±31 | 34±10 | 41±7 |
|  | Thigh | 70.6±9.7 | 2.7±0.4 | 4.3±0.6 | 572±47 | 32±3 | 5.6±0.4 | 345±28 | 339±39 | 6±16 | 44±9 |
|  | Shank | 95.6±2.5 | 4.3±0.5 | 4.5±0.5 | 657±30 | 45±3 | 6.8±0.3 | 431±18 | 378±28 | 54±19 | 61±17 |
| (iv)Body part during sideways falls | Head | 34.9±2.0 | 15.9±1.4 | 46.6±4.1 | 315±36 | 53±6 | 18.2±2.4 | 238±31 | 287±34 | -49±16 | 66±9 |
|  | Forearm | 132.3±7.8 | 36.2±3.9 | 26.7±1.5 | 1080±135 | 167±23 | 15.4±0.9 | 878±129 | 294±27 | 585±117 | 585±117 |
|  | Upper arm | 64.1±3.7 | 7.0±1.5 | 10.1±1.7 | 598±17 | 82±13 | 13.5±1.9 | 394±21 | 412±55 | -19±39 | 83±31 |
|  | Torso | 61.7±1.7 | 4.5±0.4 | 7.4±0.7 | 327±13 | 37±3 | 11.6±1.2 | 282±10 | 292±21 | -10±13 | 36±7 |
|  | Thigh | 113.8±3.8 | 45.3±3.6 | 39.8±3.0 | 1280±137 | 210±27 | 16.3±1.0 | 1038±93 | 227±22 | 810±92 | 810±92 |
|  | Shank | 86.8±1.1 | 19.9±1.4 | 23.1±1.7 | 400±24 | 73±7 | 18.6±1.6 | 339±18 | 374±26 | -35±15 | 53±9 |
| (vii) Camera angle | 90 | 79.5±2.5 | 10.1±0.9 | 13.0±0.9 | 631±23 | 63±4 | 10.0±0.4 | 462±19 | 347±11 | 115±18 | 148±16 |
|  | 60 | 79.7±2.3 | 10.6±0.7 | 15.3±1.0 | 635±26 | 74±5 | 12.0±0.5 | 471±21 | 391±17 | 79±21 | 180±18 |
|  | 30 |  | 26.6±1.9 | 40.4±2.8 |  | 121±7 | 21.6±1.5 |  | 421±25 | 50±31 | 267±25 |
| (viii) 2D calibration grid translation (averaged across forward and backward translation) | 10cm | 79.5±1.7 | 10.0±0.6 | 12.9±0.7 | 631±16 | 63±3 | 10.0±0.3 | 462±13 | 346±8 | 116±14 | 148±12 |
|  | 20cm |  | 10.1±0.6 | 13.0±0.7 |  | 63±3 | 10.0±0.3 |  | 347±8 | 115±12 | 148±12 |
|  | 30cm |  | 10.1±0.6 | 13.1±0.7 |  | 63±3 | 10.0±0.3 |  | 347±8 | 115±12 | 148±12 |
|  | 40cm |  | 10.1±0.6 | 13.0±0.7 |  | 63±3 | 10.0±0.3 |  | 346±8 | 116±12 | 148±12 |
|  | 50cm |  | 10.1±0.6 | 13.0±0.7 |  | 63±3 | 10.0±0.3 |  | 346±8 | 116±12 | 149±12 |
| (ix) 2D calibration grid rotation (averaged across clockwise and counter clockwise directions) | 15deg |  | 11.3±0.6 | 14.6±0.7 |  | 65±3 | 10.4±0.3 |  | 344±8 | 118±13 | 156±12 |
|  | 30deg |  | 13.7±0.6 | 18.3±0.9 |  | 70±3 | 11.5±0.3 |  | 364±9 | 98±13 | 164±11 |
|  | 45deg |  | 16.7±0.6 | 23.3±1.1 |  | 81±3 | 13.7±0.4 |  | 400±11 | 61±14 | 183±11 |
| (x) 2D calibration grid in the plane of the fall | | 79.5±2.5 | 10.1±0.9 | 13.0±0.9 | 631±23 | 63±4 | 10.0±0.4 | 462±19 | 347±11 | 115±18 | 148±16 |
| (xi) Forward translation of 2D calibration grid | 10cm |  | 10.1±0.9 | 13.0±0.9 |  | 63±4 | 10.0±0.4 |  | 348±11 | 114±18 | 148±16 |
|  | 20cm |  | 10.1±0.9 | 13.1±1.0 |  | 63±4 | 10.0±0.4 |  | 348±11 | 113±18 | 148±16 |
|  | 30cm |  | 10.2±0.9 | 13.1±1.0 |  | 63±4 | 10.1±0.4 |  | 348±11 | 114±18 | 149±16 |
|  | 40cm |  | 10.2±0.9 | 13.2±1.0 |  | 63±4 | 10.1±0.4 |  | 349±11 | 114±18 | 149±16 |
|  | 50cm |  | 10.2±0.9 | 13.1±0.9 |  | 63±4 | 10.1±0.4 |  | 349±11 | 113±18 | 149±16 |
| (xii) Backward translation of 2D calibration grid | 10cm |  | 10.0±0.9 | 12.8±0.9 |  | 63±4 | 10.0±0.4 |  | 344±11 | 117±18 | 148±16 |
|  | 20cm |  | 10.0±0.9 | 12.8±1.0 |  | 63±4 | 10.0±0.4 |  | 346±11 | 116±18 | 147±16 |
|  | 30cm |  | 10.0±0.9 | 13.0±0.9 |  | 63±4 | 10.0±0.4 |  | 346±11 | 116±18 | 148±16 |
|  | 40cm |  | 10.0±0.9 | 12.7±0.9 |  | 63±4 | 9.9±0.4 |  | 345±11 | 117±18 | 148±16 |
|  | 50cm |  | 10.0±0.9 | 12.8±0.9 |  | 63±4 | 9.9±0.4 |  | 343±11 | 118±18 | 148±17 |
| (xiii) Clockwise rotation of 2D calibration grid | 15deg |  | 10.4±0.9 | 12.6±0.8 |  | 63±4 | 9.8 ±0.3 |  | 328±10 | 134±18 | 154±17 |
|  | 30deg |  | 12.2±0.9 | 14.8±0.7 |  | 67±4 | 10.4±0.3 |  | 333±11 | 129±18 | 159±17 |
|  | 45deg |  | 15.0±0.8 | 19.1±0.7 |  | 76±4 | 12.0±0.3 |  | 350±13 | 112±19 | 177±17 |
| (xiv) Counter clockwise rotation of 2D calibration grid | 15deg |  | 12.2±0.9 | 16.6±1.2 |  | 66±4 | 10.9±0.4 |  | 359±12 | 103±18 | 157±16 |
|  | 30deg |  | 15.2±0.9 | 21.8±1.6 |  | 73±4 | 12.6±0.5 |  | 396±14 | 66±18 | 168±14 |
|  | 45deg |  | 18.3±0.9 | 27.6±2.0 |  | 87±4 | 15.3±0.6 |  | 451±17 | 11±19 | 189±14 |
| (xv) 1D calibration based on height | |  | 11.5±0.8 | 15.2±0.9 |  | 66±4 | 10.5±0.4 |  | 331±12 | 131±18 | 164±17 |
| (xvi) 1D calibration based on height + 10cm | |  | 11.5±0.8 | 15.2±0.9 |  | 66±4 | 10.5±0.4 |  | 331±12 | 131±18 | 164±17 |
| (xvii) 1D calibration based on height -10cm | |  | 11.5±0.8 | 15.2±0.9 |  | 66±4 | 10.5±0.4 |  | 331±12 | 131±18 | 164±17 |
