# Supplementary material for: Accuracy of Kinovea software in estimating body segment movements during falls captured on standard video: Effects of fall direction, camera perspective and video calibration technique
Source: PLoS One. 2021 Oct 25;16(10):e0258923. doi: 10.1371/journal.pone.0258923 (PMC8544843; doi:10.1371/journal.pone.0258923)
Supplement: Attachment — Submitted filename: Response to Reviewers.docx [file pone.0258923.s007.docx]

**Response to academic editor and reviewers**

We thank the editor and the reviewers for their thoughtful and valuable comments on our original manuscript. We have revised the manuscript to address the comments, and we hope you agree this has led to substantial improvements in the paper.

Below we provide a detailed response to each point raised by the editor and reviewers, including a description of corresponding changes to the manuscript. Line numbers refer to locations in the revised manuscript with tracked changes. We have attached two versions of the revised manuscript. One is a marked-up version with changes shown using “tracked changes.” The other is a clean copy where changes are not highlighted.

**Responses to Academic Editor’s comments**

**Comment 1:**

*“1. Please ensure that your manuscript meets PLOS ONE's style requirements, including those for file naming. The PLOS ONE style templates can be found at*

[*https://journals.plos.org/plosone/s/file?id=wjVg/PLOSOne_formatting_sample_main_body.pdf*](https://journals.plos.org/plosone/s/file?id=wjVg/PLOSOne_formatting_sample_main_body.pdf)

*and* [*https://journals.plos.org/plosone/s/file?id=ba62/PLOSOne_formatting_sample_title_authors_affiliations.pdf*](about:blank)*”*

**Response to comment 1:**

We reviewed the style templates and modified the manuscript to ensure it meets PLOS ONE’S style requirements. In particular, we made the following changes:

- we added a left indent to the beginning of each paragraph;

- we integrated the figure captions in the text; and

- we modified the figure citations to match the format shown in the template.

**Comment 2:**

*“2. We note that you have stated that you will provide repository information for your data at acceptance. Should your manuscript be accepted for publication, we will hold it until you provide the relevant accession numbers or DOIs necessary to access your data. If you wish to make changes to your Data Availability statement, please describe these changes in your cover letter and we will update your Data Availability statement to reflect the information you provide.”*

**Response to comment 2:**

As described in our cover letter, we wish to make changes to our Data Availability statement to solely share our data as Supporting Information Files. Therefore, we request to change our data availability statement to state that “All relevant data are within the manuscript and its Supporting Information files”.

**Comment 3:**

*“3.  We note that Figure 1 includes an image of a participant in the study.*

*As per the PLOS ONE policy (*[*http://journals.plos.org/plosone/s/submission-guidelines#loc-human-subjects-research*](about:blank)*) on papers that include identifying, or potentially identifying, information, the individual(s) or parent(s)/guardian(s) must be informed of the terms of the PLOS open-access (CC-BY) license and provide specific permission for publication of these details under the terms of this license. Please download the* [*Consent Form for Publication in a PLOS Journal*](about:blank) *(*[*http://journals.plos.org/plosone/s/file?id=8ce6/plos-consent-form-english.pdf*](about:blank)*). The signed consent form should not be submitted with the manuscript, but should be securely filed in the individual's case notes. Please amend the methods section and ethics statement of the manuscript to explicitly state that the patient/participant has provided consent for publication: “The individual in this manuscript has given written informed consent (as outlined in PLOS consent form) to publish these case details”.*

*If you are unable to obtain consent from the subject of the photograph, you will need to remove the figure and any other textual identifying information or case descriptions for this individual.”*

**Response to comment 3:**

The two individuals (the participant and the researcher) whose images are included in Figure 1 were informed of the terms of the PLOS open-access (CC-BY) license and provided specific permission for publication of these details under the terms of this license. Both individuals signed the PLOS consent forms, which were filed in the case notes.

As advised by the academic editor, we amended the Methods section and ethics statement to explicitly state (lines 273-274):

Furthermore, all individuals whose image is shown in Fig 1 have given written informed consent (as outlined in PLOS consent form) to publish these images.

**Responses to reviewer’s 1 comments:**

**Comment - Review Comments to the Author:**

*Reviewer #1: “This study sought to determine the accuracy with which an open source biomechanical software package, Kinovea, could be used to estimate the kinematics of laboratory induced falls with respect to a gold standard 3D motion capture system. This is an interesting paper, which pending revision, may lower the bar for entry into the analysis of real-world falls. This is a big positive for those of us involved in falls research. The authors therefore are to be commended for taking on this project, and doing so with such breadth of independent variables. That being said, my enthusiasm for the manuscript was somewhat tempered by a variety of concerns regarding methodology, as well as presentation and interpretation of study results. These included but were not limited to the absence of any recommendations for the use of Kinovea when analyzing falls, complex figures (which may be better suited as tables given the volume of data), and the restatement of results within the discussion.”*

**Response to Review Comments to the Author:**

We thank the reviewer for their detailed, thoughtful and constructive review. Below, we address each of the comments made by the reviewer, and highlight corresponding changes to the manuscript.

**Major comments**

**Comment 1:**

*“1. It would be extremely helpful to the reader, and ease interpretation as well as future application, if the authors provided guidelines or recommendations on the use of Kinovea for the assessment of falls videos based on the observed RMSE values (i.e., when is the RMSE ok versus "too much"?). For example, should Kinovea be used for all fall directions? Both position and velocity data? All body segments? While this is hinted at in the conclusion, it is not well described with specifics in the discussion.”*

**Response to comment 1:**

Based on this comment, and similar comments from Reviewer 2, we have modified the Abstract and Discussion to better convey recommendations on the use of Kinovea for extracting body segment kinematics from planar video of falls. Rather than providing specific threshold values for acceptability of the RMSE values, we have reframed the Discussion by stating, in the first paragraph, that for those considering the use of Kinovea for analyzing the kinematics of video-captured falls, the acceptability of the observed errors will depend on the user’s study design, and factors such as the desired detectable difference between conditions.

Specific changes are as follows.

(a) We have revised the Abstract (lines 41-45) to summarize the specific conditions where poor accuracy was observed for specific kinematic outcomes from Kinovea:

Our results demonstrate that Kinovea can be applied to 30 Hz video to measure linear positions and velocities to within 9% accuracy. Lower accuracy was observed for angular kinematics of the upper and lower limb in sideways falls, and for horizontal measures from 30 degree cameras or 1D height-based calibration

(b) We have revised the Discussion to guide the reader in understanding the observed trends in accuracy, and how these affect the acceptability of Kinovea for a given study design. Specific changes are described below.

(b.1) In the first paragraph of the Discussion, we now describe (lines 673-680):

Below, we provide guidance for those considering the use of Kinovea for analyzing the kinematics of video-captured falls. We discuss the accuracy of Kinovea observed under both “ideal” and non-ideal conditions, where ideal conditions involved: (a) the camera axis oriented perpendicular (90 degrees) to the plane of the fall, (b) the video calibrated from a 2D grid recorded in the plane of the fall, and (c) a 10 Hz cut-off frequency used for low-pass filtering of position data. We also highlight the conditions where the accuracy of specific parameters was substantially lower than values observed under ideal conditions, and discus the potential reasons for the observed trends.

(b.2) In the second paragraph of the Discussion, we describe that, for those considering the use of Kinovea for analyzing the kinematics of video-captured falls, the acceptability of the observed errors user will depend on the user’s required level of accuracy, which in turn depends on the study goals and design (for which we provide two examples). The new text reads as follows (lines 714-725):

We stress that, for a given user, the acceptability of the observed errors will depend on the user’s required level of accuracy, which in turn depends on the study goals and design. Consider, for example, a situation where a user is interested in comparing peak vertical velocities in forward versus backward falls [41], or in falls experienced by women versus men [47]. For these applications, the small error of Kinovea in measuring peak vertical velocity over a wide range of conditions should allow the user to detect small differences between conditions (if they exist). In contrast, if the user instead wishes to compare the time-varying angular velocities of body segments [48], for which Kinovea had a substantially higher measurement error, the (actual) difference between conditions may need to be quite large, to overcome the inherent errors and be detected from Kinovea-based analysis. Supplementary S1 Table allows readers to identify the expected level of accuracy for specific combinations of kinematic outcome, body part, fall direction, and approach for video collection and calibration.

(b.3) In paragraph 3, we summarize the baseline errors observed in “ideal” conditions (lines 727-730):

Under ideal conditions, Kinovea estimated vertical and horizontal positions and velocities over the duration of the fall to within 9% error, angular positions and velocities to within 13% error, and peak vertical and horizontal velocities to within 2% error and with no bias (or constant error).

(b.4) In paragraphs 4 and 5, we urge caution for some but not all body parts in relying on specific parameters in sideways and backward falls (lines 751-761):

when compared to other fall directions, Kinovea errors in sideways falls were about 4-fold higher for thigh and forearm angular position and velocity, and about 2-fold higher for knee and elbow vertical velocity. Furthermore, errors in wrist vertical velocity were about 2-fold higher in backward and sideways falls, than in forward falls. Users should be cautious in relying on Kinovea to yield accurate results for these particular combinations of fall direction and kinematic outcome.

Errors were more stable across fall directions for kinematics of the head, sternum, and pelvis. In particular, we found encouraging evidence for the accuracy of Kinovea in estimating pelvis impact velocities in sideways falls, which are relevant to the cause and prevention of hip fractures [49, 50].

(b.5) In paragraph 6, we summarize how the camera angle had little effect on the accuracy of vertical measures, but strongly affected the accuracy of horizontal and angular measures (lines 787-793):

We found that errors in vertical kinematics were relatively unaffected by camera angle, with the 30 degree camera providing less than 12% error for vertical positions and 8% error for vertical velocities. This suggests that Kinovea is appropriate for estimating vertical kinematics for a wide range of camera angles. However, the camera angle more strongly affected the accuracy of Kinovea estimates of horizontal and angular kinematics, likely due to its effect on the range of observable horizontal and angular movement. Errors in horizontal velocity were 2.5-fold higher for a 30 than 90 degree camera angle.

(b.6) Finally, in paragraph 7, we discuss the key differences in accuracy, and provide recommendations on the use of 1D versus 2D calibration (lines 899-906):

In general, there was little difference between 1D and 2D calibration in the accuracy of vertical outcomes, but errors in horizontal velocity were 1.6-fold higher for 1D than 2D calibration. However, for video collected from cameras oriented at 90 degrees, 1D calibration provided vertical, horizontal and angular velocity measures that were nearly as accurate (to within 4%) as 2D calibration values. Collectively, our results indicate that 1D video calibration based on height was nearly as accurate as 2D (grid) calibration for vertical measures, and for horizontal and angular measures when the camera was oriented at 90 degrees to the plane of the fall.

**Comment 2:**

*“2. The authors describe difficulty with differentiation of position signals to velocity data. Did the authors consider or might the consider using a Svelsky-Golay filter? We have found this quite effective in calculating velocity signals from position data with minimal noise.”*

**Response to comment 2:**

In this comment regarding the choice of filter, we assume the reviewer is referring to the following sentence in Subsection 2.4.1 of our original submission: “While not apparent in position versus time data (Fig 2A-C; left panels), numerical differentiation of unfiltered position data produced high frequency noise in velocity signals (Fig 2A-C; right panels), well above the physiological range, which was likely due to electrical noise.”

We apologize for our lack of clarity, but this statement was referring to high-frequency noise in velocity, that results from numerically differentiating position data, without any initial filtering of the position data.

We have now removed this sentence, and have replaced it with the following (lines 350-357):

To arrive at a “ground truth” set of kinematic data from Qualisys (for comparison with other signals), we identified cut-off frequencies for low-pass filtering of Qualisys position data that removed non-physiological high frequency noise. From Fourier analysis, we found that less than 4% of the signal energy content was above 20 Hz, for 96.7% of all unfiltered vertical velocity traces (n=365). Accordingly, Qualisys ground truth signals were based on position data processed with a 4th order, dual-pass Butterworth low-pass filter using a 20 Hz cut-off frequency. Visual inspection confirmed that the 20 Hz low-pass filter preserved the physiological components of the movement (Fig 2A-C).

We agree that different types of filters (e.g., Savitzky Golay) may be useful, but found no drawbacks in applying a fourth-order Butterworth which provided a convenient means for examining the effect of cut-off frequency as a proxy for camera frame rate.

**Comment 3:**

*“3. Figures 4 through 8 are a lot to take in. Given the density of the data would this data be better presented as tables?”*

**Response to comment 3:**

After carefully considering this comment, we have decided to retain Figures 4-8, and complement these figures with a table that provide the same data, provided as supplementary material (S1 Table). We agree the figures contain large amounts of data. We worked to create various tables that could replace the figures, but ultimately found that, due to the large number of signals for comparison and level of our independent (explanatory) variables, the tables were less clear for conveying the results. The S1 Table provides RMSE values, as well as signal amplitudes and normalized RMSE, for vertical, horizontal and angular outcomes. The tables include values for all levels of explanatory variables shown in Figures 4-8, including cut-off frequency, fall direction, body part, camera angle, and calibration technique. With this addition, the results are available to readers as both figures and tables, with p-values for the comparisons provided in the figures.

The revised legend for S1 Table reads as follows (lines 1177-1231):

**S1 Table. Differences in positions and velocities between Qualisys ground truth signal and Kinovea signals.** The table shows differences between Qualisys ground truth signal (Q) and Kinovea signals (K) in (A) vertical, (B) horizontal and (C) angular position and velocity signals. Comparisons show the effects of different (i) cut-off frequencies, (ii) fall directions, (iii-vi) body parts for each fall direction, (vii) camera angles, and (viii-xvii) calibration techniques and orientations. In the camera angles comparisons, Qualisys values (vertical, horizontal, and angular) are different between the 90 degree, and the 60 and 30 degree cameras because markers from the right versus the left side of the body were analyzed, respectively. RMSE and NRMSE are based on comparing signals throughout the fall. Results show mean ± 1 SE values for all falls in a given category. For positions, values less than 0.001 m are rounded up. For velocities, values less than 0.01 m/s are rounded up.

**Comment 4:**

*“4. Much of the discussion, and especially the first 2-3 sentences of nearly every paragraph, consists of a restatement of the results. This seems unnecessary. The discussion could be better focused on how and when to use Kinovea for the analysis of falls based on comment 1 above. This may align the discussion with the results quite well.”*

**Response to comment 4:**

We appreciate this comment, and have worked to revise the Discussion to remove redundancy (in restating the Results), and to focus more on the issue of how and when to use Kinovea for the analysis of falls. We refer the reviewer to our response to Comment 1, where we describe changes and provide quotes from the revised text.

**Comment 5:**

*“5. Could the authors provide a justification for the use of Kinovea versus some other open source software package like DeepLabCut that has become popular for the analysis of video data. Is a comparison of Kinovea to DeepLabCut necessary?”*

**Response to comment 5:**

We acknowledge that other available software packages, such as DeepLabCut and OpenPose, provide an alternative to Kinovea for the analysis of video footage of falls. We have now addressed this issue in the limitations section of the Discussion (lines 923-924) as follows:

Finally, studies are needed to compare the accuracy of Kinovea to alternative approaches for extracting kinematics from planar video (e.g., DeepLabCut [53]).

**Minor comments**

**Comment 1:**

*“1. Could the authors clarify why they chose to calculate segment rather than anatomical joint angles?”*

**Response to comment 1:**

We agree on the relevance of this question, and on now clarify in the Methods (on lines 390-392) why we focused on segment angles rather than relative (joint) angles, as follows:

We focused on segment angles; rather than relative (joint) angles, since falls researchers are often interested in the orientation of body segments with respect to the vertical or the ground [45, 46].

**Comment 2:**

*“2. Could the authors clarify whether reflective markers used in the 3D analysis were or were not used when processing the Kinovea data? If so, what implications might this have for the application of Kinovea to video without passive reflective markers?”*

**Response to comment 2:**

We agree this is an important question, and we now have added the following text in the limitations section in the Discussion (lines 916-918):

Second, when visible in the video footage, the reflective markers used for 3D motion capture helped in identifying body part locations to track in Kinovea. The lack of distinct landmarks on body parts in real-life falls may affect the accuracy of the measurements.

**Comment 3:**

*“3. In the methods the authors describe an assessment of inter-rater reliability using RMSE. It is not immediately clear if this is an appropriate method by which to assess inter-rater reliability. Should the authors be determining inter-rater reliability using intra-class correlation coefficients (ICC)? The following citation may provide guidance in this decision.*

*Weir JP. Quantifying test-retest reliability using the intraclass correlation coefficient and the SEM. J Strength Cond Res. 2005;19(1):231-240.”*

**Response to comment 3:**

We thank the reviewer for their comment and advice on how to best describe inter-rater reliability from the two raters. We have revised subsection 2.4.4 to report both RMSEs between the raters (to reflect the magnitude of the difference) and intra-class correlation coefficients (ICC). The modified text reads as follows (lines 408-417):

We examined the inter-rater reliability of kinematic signals from Kinovea by comparing results from two raters, who independently digitized and calibrated videos of whole-body movements from three perturbation-based falls. RMSEs in linear position and velocity throughout the fall were compared for all camera angles, along with Intraclass Correlation Coefficients (ICC) (SPSS for Windows, version 25, IBM Corp., Armonk, N.Y., USA).

The mean RMSE between two raters was 0.011 m (SE = 0.001) for position, or 2.4% (SE = 0.2) of the signal amplitude, and 0.13 m/s (SE = 0.01) for velocity, or 4.8% (SE = 0.3) of the signal amplitude. ICC values and 95^th^ confidence intervals were 1.00 (1.00-1.00) for linear position and 0.985 (0.984-0.986) for linear velocity.

**Responses to reviewer’s 2 comments:**

**Comment - Review Comments to the Author:**

*“Reviewer #2: Accuracy of Kinovea software in estimating body segment movements during falls captured on standard video: effects of fall direction, camera perspective and video calibration technique*

*Summary of reviewer comments: The manuscript focuses on a significant and clinically-relevant problem –measurement of movement quality during falls in real-world settings. The study methods comprised laboratory-based falling experiments, and comparison of full body kinematics (linear and angular positions and velocities) measured from 3D motion capture to those estimated by Kinovea 2D digitization software from standard surveillance video cameras. The effects of several methodological factors on the accuracy of Kinovea were evaluated, including fall direction, camera angle, filtering cut-off frequency, and calibration techniques. The results are comprehensive and presented clearly. However, the rationale for the study and comparison of current work in light of previous literature needs strengthening. The study is limited by a very small sample size (N=3), which may be caused by the unique methodology employed but needs justification. The discussion can benefit from reorganization and editing.”*

**Response - Review Comments to the Author:**

We thank the reviewer for their detailed, thoughtful and constructive review. Below, we address each of the comments made by the reviewer, and highlight corresponding changes to the manuscript.

**Comment - Abstract:**

*“Abstract – The statements regarding the results can benefit from inclusion of some numbers, e.g. “When compared to 90 deg, a 60 deg camera angle yielded less accurate horizontal velocities (include magnitude or % decrease in error here). The conclusion of the abstract could be edited to be more specific and to better discuss the implications of the results.”*

**Response to abstract comment:**

Based on this comment, and a similar comment from Reviewer 1, we have modified the Abstract to quantify the size of the observed differences in accuracy, and discuss the implication of the results. Specific changes are as follows.

(a) We have revised the Abstract (lines 37-41) to describe the size of the observed differences in measurement accuracy:

Errors in angular measures averaged over 2-fold higher in sideways than forward or backward falls, due to out-of-plane movement of the knees and elbows. Errors in horizontal velocity were 2.5-fold higher for a 30 than 90 degree camera angle, and 1.6-fold higher for calibration using participants’ height (1D) instead of a 2D grid. When compared to 10 Hz, filtering at 3 Hz caused velocity errors to increase 1.4-fold.

(b) We now conclude the Abstract (lines 41-45) with specific recommendations on the use of Kinovea for the assessment of falls videos:

Our results demonstrate that Kinovea can be applied to 30 Hz video to measure linear positions and velocities to within 9% accuracy. Lower accuracy was observed for angular kinematics of the upper and lower limb in sideways falls, and for horizontal measures from 30 degree cameras or 1D height-based calibration

**Comments - Introduction:**

**Comment 1:**

*“Introduction –*

*“Improved understanding of the kinematics of real-life falls should help to inform efforts for injury prevention” – it would strengthen the introduction if the authors could provide details or examples of how measuring kinematics during falls can inform fall prevention programs.”*

**Response:**

We appreciate this comment, and have revised the second paragraph of the Introduction to provide details or examples of how measuring kinematics during falls can inform fall prevention programs, as follows (lines 187-193):

Improved understanding of the kinematics of real-life falls should help to inform efforts for injury prevention. For example, information on the movements of the body during falls can help exercise therapists in designing exercise programs for fall prevention [7-10], engineers in creating mobility aids [11-13] and architects and designers in creating safer environments [14-17]. Data on the kinematics of falls can also guide the design of improved sensor-based automatic fall detection systems [18-23]. Furthermore, data on the impact velocities of the body during falls is essential for the design and evaluation of fall protective gear [24-28] and compliant flooring.

**Comment 2:**

*“compared to 3D motion capture, Kinovea estimated hip, knee, and ankle angles during walking with errors less than 5 deg” – please clarify if this stated accuracy or error is for 2-D or sagittal plane angles? Also, please refrain from using “deg” instead of degrees throughout the text of the manuscript.”*

**Response:**

Thank you for pointing out the need for clarification on whether the stated accuracy is for 2-D or sagittal plane angles. We now address this question through the following revision to the text (lines 218-220):

When compared to 3D motion capture, Kinovea estimated hip, knee, and ankle angles in the sagittal plane during walking with errors less than 5 degrees [42], and sagittal-plane knee angles during drop jumps with less than 10% error [43].

Furthermore, we have changed all appearances of “deg” to “degree” throughout the text of the manuscript.

**Comment 3:**

*“Because the manuscript has several objectives related to evaluation of Kinovea for measurement of kinematics during falls, it would improve clarity if the authors could edit the last para of the Introduction to clearly list the study objectives (in order of importance), and also provide the rationale or importance of each stated objective. Additionally, if possible, a couple sentences about previous studies that may have addressed the same or similar questions would be beneficial for the reader.”*

**Response:**

We appreciate this comment, and have revised the last paragraph of the Introduction to clearly list the study objectives in order of importance, and to provide the rationale or importance of each stated objective. We have also referred to previous studies that addressed similar questions. The revised paragraph reads as follows (lines 222-265):

In the current study, we examined the accuracy of Kinovea for estimating the kinematics of falls from planar video with a resolution (640 x 480 pixels) and frame rate (30 Hz) typical of that acquired by surveillance video cameras. We addressed four objectives. First, we sought to determine the accuracy of Kinovea estimates under ideal video recording conditions, involving the camera axis oriented perpendicular to the plane of the fall [44], and calibration of the video (in m/pixel) based on a 2D grid recorded in the plane of the fall. We measured accuracy by comparing estimates from Kinovea to measures from high speed, 3D motion capture of the linear and angular positions and velocities of the head, torso, pelvis, upper limbs, and lower limbs. Second, we sought to determine how the accuracy of Kinovea was affected by the direction of the fall, and confirm whether our results support previous observations of good accuracy in kinematic estimates from planar video for backward falls [40], and higher errors for sideways falls, due to out-of-plane movements of the body segments [41]. Third, we examined how the accuracy of Kinovea is affected by the camera angle and frame rate (simulated through low-pass filtering the position data), which may vary widely in real-life conditions [35]. Fourth, we examined the accuracy of a simple 1D calibration approach based on the height of the faller, as a practical alternative to a 2D calibration grid in the plane of the fall. Our results provide a basis for understanding what can be accurately measured, and for recommended approaches for using Kinovea to analyze the kinematics of falls from 2D planar video.

**Comments – Methods:**

**Comment 1:**

“Methods –

*The study involves a very small sample size (3 individuals). Please clarify the rationale for this sample size in the methods section, and a statement about the sample size as a limitation in the Discussion section.”*

**Response:**

We appreciate this comment, and have now revised the Methods and the limitations section of the Discussion to clarify the rationale of our sample size of n=36 falls collected from three participants. Specific changes are described below.

(a) In the subsection 2.1 of the Methods section (Participants), we now describe (lines 269-270):

Thirty six falls were performed by three young adults who participated in this study (two women aged 32 and 22 years, and one man aged 34 years)

(b) In the subsection 2.5 of the Methods section (Statistical analysis), we describe (line 481):

Each fall was treated as an independent measure.

(c) In the limitations section of the Discussion, we now state (lines 908-910):

First, all of our data was collected from three healthy young adults, who experienced falls onto a firm gym mat, that were either self-initiated (simulating incorrect weight shifting), or caused by rapid translation of the ground surface.

**Comment 2:**

*“The methods used for the 2 different camera calibration methods should be described in more detail, and appropriate supportive references provided, if available.”*

**Response:**

We agree on the value of providing additional details on the nature of the two approaches used in calibrating the video footage, and have modified section 2.4.5 in the Methods as follows (lines 419-436):

## 2.4.5 Effect of cut-off frequency and calibration technique on accuracy of Kinovea data

## We examined the accuracy of Kinovea data for cut-off frequencies of 3, 5, 7, 10, 12, and 14 Hz for low-pass filtering of position data. 14 Hz was selected as the maximum cut-off frequency for filtering the 30 Hz data, based on Nyquist considerations. We also examined how the accuracy of Kinovea outcomes differed between two approaches available in Kinovea for calibrating the videos (i.e., converting from pixels to m): a 2D grid versus a 1D vertical line (Fig 1C). The 2D grid was of dimensions 1.6 x 1.6 m, and contained a 5 x 5 array of dots spaced 40 cm apart. At baseline, the grid was located in the plane of the fall. The 1D vertical line was placed in a frame when the participant was standing prior to the onset of the fall. The line extended from the base of the foot to the top of the head, and was set equal to participant height. We also examined how signal accuracy was affected by: (a) translating the grid by ±10, ±20, ±30, ±40, and ±50 cm from the plane of the fall, where negative and positive values reflect translation away from and towards the camera, respectively (Fig 1D); (b) rotating the grid ±15, ±30, and ±45 degrees from the plane of the fall (Fig 1E); and (c) introducing errors in participant height of ±10 cm from the actual values.

**Comment 3:**

*“There may be a formatting error in Table 1 (columns on the far right appear truncated/cropped).”*

**Response:**

Indeed, Table 1 contains columns on the far right that extend beyond the page margin, which can be viewed in Word using Draft View. We apologize if this creates challenges in the review process. We have followed the recommendations of the journal (<https://journals.plos.org/plosone/s/tables>) which specify: “Tables do not have strict width and height requirements. Do not split your table or otherwise try to make the table appear within the manuscript margins if it does not fit on one page. In Word, tables that run off of the manuscript page can be seen using Draft View. In the PDF version of the published article, very wide tables may be printed sideways, and long tables may span more than one page.”

**Comment 4:**

*“We used one-way ANOVAs to examine how the accuracy of Kinovea signals associated with Kinovea filter cut-off frequency, camera angle, fall direction, body part, and calibration technique”- this description is somewhat confusing as written. Was the ANOVA used to test associations or the effect of these methodological variables on errors? Were separate ANOVAs performed for each variable (e.g. camera angle, filter settings, etc)? Please edit to improve clarity. Similarly, the rationale for repeated measures comparisons for the same fall could be better clarified.*

**Response:**

We agree on the value of improved clarity on these issues. We have now modified the statistical analysis section to: (a) clarify that the ANOVAs were used to test the effect of the methodological variables on errors; (b) clarify that separate ANOVAs were performed for each variable (filter cut-off frequency, camera angle, fall direction, body part, and calibration technique); and (c) provide an improved rationale for using repeated measures comparisons for all variables (expect fall direction). The revised text reads as follows (lines 478-488):

# **2.5 Statistical analysis**

We used one-way ANOVAs to examine the effect of the following methodological variables on the accuracy of Kinovea signals: Kinovea filter cut-off frequency, camera angle, fall direction, body part, and calibration technique. Each fall was treated as an independent measure. Fall direction was analyzed with independent samples ANOVAs because the comparisons were between different falls. All other variables were analyzed with repeated measures ANOVAs (with the fall treated as a random factor) because these variables related to different approaches (or treatments) for collecting or processing data from the same set of falls. In case of significant main effects, Tukey’s Post hoc analysis was used to test pair-wise comparisons. All ANOVA analyses were conducted using JMP software (Version 15. SAS Institute Inc., Cary, NC) using a significance level of α<0.05.

**Comments – Results and Discussion:**

**Comment 1:**

*“Results and Discussion –*

*The Discussion can be strengthened by including more detailed interpretation of the study findings, placing the current results in context of previous literature, as well as methodological and clinical implications or insights gained from the study.”*

**Response:**

We agree with this comment, and have revised sections of the Discussion to provide a more detailed interpretation of the study findings in the context of previous literature, and a description of the implications or insights gained from the study. Specific changes are described below.

(a) In paragraph 5 of the Discussion, we discuss discrepancies between our results and those reported by Choi et al. for peak pelvis velocities from digitizing video footage of sideways falls (lines 759-783):

we found encouraging evidence for the accuracy of Kinovea in estimating pelvis impact velocities in sideways falls, which are relevant to the cause and prevention of hip fractures [48, 49]. Unlike Choi et al. [41], who observed errors that they deemed as unacceptably high for peak pelvis velocities from digitizing planar video of sideways falls (mean error = 1.01 m/s (SD=0.59) for vertical velocity, and 0.13 m/s (SD 0.54) for horizontal velocity), we found that Kinovea estimated peak velocities of the ASIS during sideways falls to within an error of 0.12 m/s (3.9%) for vertical velocity, and 0.14 m/s (10.7%) for horizontal velocity. It is difficult to explain why the errors observed by Choi for sideways falls were similar to ours for peak horizontal pelvis velocity, but much higher for peak vertical pelvis velocity, but this may relate to Choi et al. examining only a single fall in each of the forward, sideways and backward directions, while we analyzed 12 falls in each direction.

(b) In paragraph 6 of the Discussion, we describe how our results on the effect of camera angle compare to others (lines 795-798):

Our results agree with previous studies which reported that 90 degree camera angles provided the most accurate horizontal and angular outcomes for Kinovea analysis of body movements in hockey [51], simulated walking [44], and single leg squats [52].

(c) In paragraph 2 of the Discussion, we discuss the implications of our results to falls research. In particular, we describe that, for those considering the use of Kinovea for analyzing the kinematics of video-captured falls, the acceptability of the observed errors user will depend on the user’s required level of accuracy, which in turn depends on the study goals and design (for which we provide two examples). The new text reads as follows (lines 714-725):

We stress that, for a given user, the acceptability of the observed errors will depend on the user’s required level of accuracy, which in turn depends on the study goals and design. Consider, for example, a situation where a user is interested in comparing peak vertical velocities in forward versus backward falls [41], or in falls experienced by women versus men [47]. For these applications, the small error of Kinovea in measuring peak vertical velocity over a wide range of conditions should allow the user to detect small differences between conditions (if they exist). In contrast, if the user instead wishes to compare the time-varying angular velocities of body segments [48], for which Kinovea had a substantially higher measurement error, the (actual) difference between conditions may need to be quite large, to overcome the inherent errors and be detected from Kinovea-based analysis. Supplementary S1 Table allows readers to identify the expected level of accuracy for specific combinations of kinematic outcome, body part, fall direction, and approach for video collection and calibration.

**Comment 2:**

The discussion should also include potential reasons for instances where fairly high errors were observed (e.g. Figure 7 C shows high angular position errors for certain segments).

**Response:**

We appreciate this comment, and we have revised sections of the Discussion to focus on the potential reasons for instances where high errors were observed, as described below.

(a) In paragraph 3 of the Discussion, we discuss the potential causes for errors in Kinovea estimates under ideal conditions (lines 727-733):

Under ideal conditions, Kinovea estimated vertical and horizontal positions and velocities over the duration of the fall to within 9% error, angular positions and velocities to within 13% error, and peak vertical and horizontal velocities to within 2% error and with no bias (or constant error). These baseline errors were likely due to human inaccuracy in digitization of the video footage with Kinovea, and perhaps the limited (640 x 480) resolution of the video. It is unlikely that the errors arose from systemic causes (since Kinovea was just as likely to overestimate as underestimate peak velocities), or from limitations related to the 30 Hz video frame rate.

(b) In paragraph 4 of the Discussion, we discuss how errors in Kinovea estimates were caused by out-of-plane movements of the knee, elbow and wrist in sideways and backward falls (lines 747-754):

We found that the accuracy of Kinovea depended on fall direction. Since Kinovea involves a 2D analysis of planar video, its accuracy will be reduced by out-of-plane movements of body segments (orthogonal to the plane of the video image). From our Qualisys 3D measures, out-of-plane movements were largest at the knee and elbow in sideways falls, and at the wrist in backward falls. This explains why, when compared to other fall directions, Kinovea errors in sideways falls were about 4-fold higher for thigh and forearm angular position and velocity, and about 2-fold higher for knee and elbow vertical velocity. Furthermore, errors in wrist vertical velocity were about 2-fold higher in backward and sideways falls, than in forward falls.

(c) In paragraph 6 of the Discussion, we discuss the reasons why camera angle affected the accuracy of horizontal and angular measures (lines 790-792):

… the camera angle more strongly affected the accuracy of Kinovea estimates of horizontal and angular kinematics, likely due to its effect on the range of observable horizontal and angular movement.

**Comment 3:**

*“Figure 1 could benefit from additional labels to show the relevant objects / landmarks in each panel.”*

**Response:**

We agree with of this suggestion, and have now added labels to Figure 1. We have also modified the caption of Figure 1 to clarify the anatomical landmarks, as follows (lines 290-299):

**Fig 1.** **Experimental setup** Experimental setup for (A) forward and backward and (B) sideways falls illustrating Kinovea traces from digitization of key anatomical locations. Right panels display digitized traces from Kinovea of the time-varying positions during the falls of the head (pink), shoulder (green), elbow (purple), wrist (yellow), hip (light blue), knee (red), ankle (grey) and sternum (dark blue). Pink tape of the mat displays the plane of the fall. We examined how the accuracy of kinematic outcomes from Kinovea depended on (C) calibration technique (2D calibration grid versus 1D calibration based on participant height), (D) calibration frame translation (purple grids for forward falls and green grids for backward falls), (E) calibration grid rotation (blue grids), and camera view with respect to the fall (30, 60 and 90 degree views shown in (A), (C), and (B), respectively).
